# COVID-19 and (ir)responsible mobility:　Reading counter-practices through Derrida

**DOI:** 10.12688/openreseurope.16686.1

**Published:** 2024-01-03

**Authors:** Raffaela Puggioni

**Affiliations:** 1 Jindal School of International Affairs, OP Jindal Global University, Sonipat, Haryana, 131001, India

**Keywords:** Civic responsibility, lockdown, immobility, sans-papiers, Paris, ethics

## Abstract

During the COVID-19 pandemic, issues of civic (ir)responsibility were often raised when people broke immobility rules. Despite widespread public debate, the issue of responsibility has attracted little academic attention, as if to act responsibly simply meant obeying dominant (illiberal) norms. This article critically investigates the relationship between mobility and (ir)responsibility. It engages with the following questions: is mobility during a time of forced immobility always an irresponsible act? Should (ir)responsibility also take into account the motives for mobility? To what extent does assisting or making visible those in need transform mobility into responsible mobility? These questions are considered in the context of certain mobility counter-practices adopted in Paris to cater to the needs of marginalised and invisible groups. In particular, this article investigates responsibility and irresponsibility through the lenses of Jacques Derrida’s work, which reads the two terms as inherently interlinked, because any decision on how to act necessarily encompasses both.

## Introduction

During the COVID-19 pandemic, many nations introduced mobility restrictions and made some protective measures compulsory, such as quarantine, stay home policy, mask wearing and social distance. In European Union (EU) countries, freedom of movement was severely restricted from March 2020 up to at least until the end of 2022, even if, in reality, lockdown measures were in place, on average, for no more than six to eight months. It would not be an exaggeration to claim that many, irrespective of the country they were living in, experienced some level of anxiety, insecurity, grief, helplessness and reduced freedom ( Fitzgerald *et al.*, 2021; Szabo *et al.*, 2020; Yıldırım *et al.*, 2022). This was especially true during complete lockdown, which generally coincided with skyrocketing levels of infection and high death rates. EU countries, for the most part, adopted similar health policies and practices — for instance, stay-home policies, quarantine requirements, mask mandates and mobility restrictions. Apart from Sweden—which relied mostly on ‘voluntary compliance’ ( Larsson 2022; see also Kamerlin & Kasson, 2020; Kavaliunas *et al.*, 2020; Kuhlmann *et al.*, 2021)—EU governments introduced a variety of technologies of control to ensure compliance with the new rules of conduct ( Bigo *et al.*, 2021; Celermajer & Nassar, 2020; Eck & Hatz, 2020). Fines and penalties were imposed in case of non-compliance, particularly disrespect of mobility restrictions. Unauthorised movement was mostly read as irresponsible, because by violating mobility restrictions, people potentially jeopardised not only their own lives, but also the well-being of the population at large. Immobility, social distancing and new hygienic practices were deemed key tools for protecting people from contagion. Protection and care for those in need was done at a distance ( Tomasini, 2021). Although questions surrounding civic responsibility have frequently been raised in public debates—as many governments highlighted the need for people to act responsibly by strictly conforming to COVID-19 restrictions—the issue of (ir)responsibility has not attracted much academic attention. The question of what constituted a responsible citizen during the pandemic was somehow taken for granted, as if strict obedience was the only option. Civic attitudes have mostly been scrutinised in terms of political activism. This has been done either in investigations of social movements—particularly street protests, collective mobilisation and the strategies and motives of protests (see Kowalewski, 2021; Kriesi & Oana, 2022; Neumayer *et al.*, 2023; Pleyers, 2020; Plümper *et al.*, 2021; Pressman & Choi-Fitzpatrick, 2021; Zajak *et al.*, 2020)—or by questioning the state of Western democracy (see Afsahi *et al.*, 2020; Celermajer & Nassar, 2020; Degerman *et al.*, 2023; Greitens, 2020). The matter of whether a national or international emergency can transform the concept of the good and thus the responsible citizen has rarely been discussed.

This article examines the concept of (ir)responsibility in relation to mobility. However, rather than investigating how people resorted to everyday mobility practices to (re)establish some level of normalcy, as done in previous works ( Puggioni, 2022; Puggioni, 2023a), this article scrutinises the tension between following dominant rules and evading them in order to assist those in need. More specifically, it questions the followings: what does it mean to act responsibly during a life-threatening emergency? To what extent is mobility—during a time of forced immobility—an irresponsible act? How can ethically driven mobility be judged? What criteria can we use to evaluate (ir)responsible acts? Should they be based on their conformity (or lack thereof) to dominant rules? Or according to the ethics underlying those acts?

By focussing on the creative modalities through which people in Paris circumvented mobility restrictions, this paper suggests reading such acts of mobility as *ethically* responsible. This is because the aim of these acts was to assist or make visible those in conditions of (extreme) need. They were, at the same time, *legally* irresponsible because they occurred in contravention of the spirit and letter of the restrictions. The tension between acting responsibly towards some and irresponsibly towards others is discussed using the work of Jacques Derrida, who highlights the aporia inherent in the concept of responsibility. For Derrida, (ir)responsibility emerges not simply as ‘a dissident and inventive rupture with respect to tradition, authority, orthodoxy, rule, or doctrine’ ( 1995a: 27), but as an *intentional* rupture, always dictated by ethical concerns. This thinking also seems to apply to the narratives explored in this article.

The argument is developed in four steps, looking at 1) the mobility restrictions in France; 2) the conditions of confinement and invisibility experienced by asylum-seekers ^1^ and *sans-papiers* during the first months of the COVID-19 emergency; 3) mobility counter-practices in Paris, and 4) Derrida’s conception of (ir)responsibility.

## Methods

The overall aim of the MOBILISE project—upon which this article is based—was to investigate and make sense of instances of mobility during COVID-19 restrictions. When carrying out the desk research on the French experience, I realised that a focus on the Black Lives Matter protests—as originally planned—would not add any major innovative element, given the great amount of attention it has already received. Moreover, given my expertise on mobile subjects’ counter-practices ( Puggioni, 2005; Puggioni, 2014a; Puggioni, 2014b; Puggioni, 2018), I re-evaluated my research trajectory and decided to devote greater attention to the experiences of asylum-seekers and *sans-papiers*, the most marginalised and invisible group throughout the pandemic.

During my desk work, ^2^ I collected information on COVID-19 restrictions in France by accessing official reports, interviews, and legal documents available at the National Assembly, Ministry of the Interior, Ministry of Solidarity and Health, and Police Department ( *Préfecture de police*). I investigated the living conditions of the *sans-papier* in Paris by searching both academic articles and newspaper articles. This was done using both Google Scholar and Google search. Special attention was given to the news published in the followings: *Liberation*, *France24*, *Le Monde* and *L’Humanité*. To identify the sources, I used the following keywords, or a combination of them: *sans-papiers* in Paris, COVID-19, public gathering, public protests, acts of solidarity, responsibility, civism, camps, confinement, police controls, and Coordination Sans Papiers 75.

In terms of the research framework, I adopted a grounded theory approach, even if only three interviews have been carried out. It was, especially, the narratives of my respondents that made me change perspective. My attention shifted from public, and visible, events—organised despite COVID-19 restrictions—to everyday strategies of civic and political engagement. ^3^ Only after a careful reading and analysis of the narratives collected, I considered articulating their acts of mobility as “ethically driven counter-practices”. This was possible thanks to my respondents, who did not simply answer my few questions, ^4^ but they also shared *their* (embodied) experience of the pandemic. It was, especially, the narratives of two of them which made me rethink civic engagement, not through the “liberal subjects” prism, but through the responsibility prism. It was only at this point that I introduced Jacques Derrida’s work on ethics as a key theoretical tool for engaging more deeply with the issue of (ir)responsible mobility.

By the “liberal subjects” prism, I refer to the liberal attitude that people continued to adopt despite (severe) restrictions. As discussed in my previous work ( Puggioni, 2022), I assume that European citizens—irrespective of the restrictions enforced on them in the attempt to contain the virus—did not abandon their liberal approach to life. In other words, despite states declaring a state of emergency, European citizens did not transform themselves—nor did EU governments intend to effect such a change—into docile and obedient bodies ( Foucault, 1995) at the mercy of governmental (illiberal) decisions. They remained not only *liberal* subjects, but also *responsible* for their personal choices, including those that risked contagion by ignoring health-related restrictions, organising public protests and refusing vaccinations. Without freedom, ethics itself is unachievable, as Michel Foucault already highlighted during an interview in 1984, recognising that ‘Liberty is the ontological condition of ethics’ ( Fornet-Betancourt *et al.*, 1987: 115).

A final methodological note is necessary. Given the difficulties associated with uncovering everyday ethical practices—which mostly go unnoticed and unrecorded—and the limited number of interviews I was able to carry out, I recognise that making any generalisations based on this research is problematic. Nevertheless, the research findings of this article will, hopefully, encourage a (theoretical) debate on questions of *ethical* and *civic* responsibility during a life-threatening emergency.

## COVID-19 restrictions in France: An overview

In France, on 23rd February 2020, phase one of the COVID-19 containment began. On the 29th, public gatherings of more than 5000 people were banned; this figure was reduced to 1000 on 9th March. A few days after, on the 12 ^th^, President Emmanuel Macron announced the closure of all nurseries, schools and universities, and non-essential companies were asked to conduct their work using online platforms ( APUR, 2021: 6). Notwithstanding the health crisis, the first round of municipal elections was held in 35,000 municipalities on 15th March. ^5^ Two days after, on the 17 ^th^, at noon, lockdown measures were announced, and a state of health emergency ( *l*’ *état d’urgence sanitaire*) was officially declared ( Assemblée Nationale, 2020).

The first restrictive measures, initially introduced for 15 days, were prolonged—as was the case in other EU countries—until 10th May, which marked the beginning of phase one of the lifting of restrictions. During the 55 days of lockdown, as the *L’Humanité* (2020) puts it: ‘the whole of France lived under a bell’. ^6^ As in most EU countries, the end of lockdown measures simply meant the relaxation of the most restrictive norms, not the end of the health emergency. For instance, in the 24 hours that preceded citizens’ “exit” from confinement, on 11th May, 70 people were reported to have died due to COVID-19. The situation was not, however, the same everywhere in France. As Prime Minister Edouard Philippe clarified during a press conference on 7th May, the process of *déconfinement* was the first step in fighting the epidemic; this was to last a few weeks, until 2nd June. Greater relaxation was permitted in the ‘green’ zones, which were reporting a lower number of infections and hospitalisations, as compared to the ‘red’ zones—for instance, Île-de-France. However, the prime minister still recommended ‘to keep observing, on a voluntary basis, very strict rules of caution’ ( Vie Publique, 2020). As in other EU countries, mask-wearing on public transportation and in shops was made compulsory, as was social distancing. The ban on public gatherings was retained. Between March and May, adjustments to the restrictions were constantly made, according to the epidemiological curve (see APUR, 2021: 6–7).

During the lockdown, mobility was permitted only in certain circumstances, and people had to always carry with them a self-certificate declaring their intended destination and reason for being out—the so-called *attestations de déplacement dérogatoire*. People could leave their homes only if they worked in essential sectors—such as transportation, education, health or the food industry—or needed to buy basic necessities or engage in individual physical activities. Those caught outside without proper justification could be subjected to a fine. This was initially set at EUR 35 and was subsequently increased to EUR 135 on 18th March but could also go up to EUR 375 ( France24, 2020a). On the first day of the lockdown, dissuasive mechanisms were put in place: around 100,000 gendarmes and police were deployed to enforce the lockdown measures, and about 4095 people were found violating the new rules ( France24, 2020b).

According to Jérémie Gauthier, the controls were especially harsh in working-class neighbourhoods ( *quartiers populaires*)—with a high concentration of migrant population—that were ‘under police pressure’ ( 2020: 57). Indeed, the controls reinforced ‘discriminatory and racist treatments’ (57). Gauthier is especially critical of the Ministry of the Interior’s approach and the ways in which controls were implemented. In light of the official figures—provided by Minister of the Interior, Christophe Castaner—Gauthier points out that fines were disproportionately imposed on people living in poor areas, which also registered higher infection rates than other areas ( 2020: 59–60). Didier Fassin is also critical of the *inégalité* perpetuated during the COVID-19 pandemic in France. In his article, *L’inégalité des vies en temps d’épidémie* ( 2020), published in the left-wing newspaper *Libération*, he highlights in particular the poor living conditions inside prisons and the high risk of contagion in tight and overcrowded spaces.

Mobility restrictions—generally referred to as *mesures barrières*—and social distancing requirements remained in place until the end of August 2020. As specified in the general norms that the Ministry of Solidarity and Health ( Ministère des solidarités et de la santé, 2020) issued on 1st June, one-metre physical distancing was to be maintained ‘in all circumstance[s]’ (art. 1.I); public gatherings, reunions or any other activity taking place in a public venue, with more than 10 people present, were ‘prohibited throughout the territory of the Republic’ (art. 3.I). Article 3.V further stated that public events with more than 5000 people were banned until the end of August, and that the local prefect (the local security authority) was in charge of authorising public events depending on the prevailing local conditions and possible risks (art. 3.IV).

Vulnerabilities existed among certain groups of people in virtually all countries, and France was certainly not an exception. For instance, in both the USA and the UK, minority groups— especially Black and Asian people—were associated with high contagion and mortality rates, as well as the largest share among those working in essential capacities ( Laurencin & McClinton, 2020). Although (forced) confinement was challenging for citizens worldwide, some groups were exposed to greater deprivation, restrictions, job insecurity or loss, deterioration of their economic condition and risk of contracting the virus ( Beaman, 2020; Crouzet *et al.*, 2022; Zaouche Gaudron *et al.*, 2022). Among the disadvantaged, asylum-seekers and *sans-papiers* were probably the worst off. It is to them that I now turn my attention.

## The ‘uncounted’ ^7^

In Paris, during the COVID-19 pandemic, the living conditions of asylum-seekers and *sans-papiers* were extremely precarious (as is still the case), and the majority of unaccompanied minors were abandoned. As soon as ‘The Jungle’—the unauthorised camp in Calais—was closed down in 2016, Paris became the new destination. According to Madeleine Byrne (2021), approximately 3000 asylum-seekers, refugees and *sans-papiers* moved into informal camps in the city’s northern areas. In recent years, these people have made themselves visible by pitching tents in key Parisian squares or outside well-known buildings, such as Place de la République, Panthéon, Charles-de-Gaulle Airport, France’s Court of Cassation and Place Saint-Gervais (see Taskin, 2022). Such acts of visibility do not normally last long as the police not only forcibly remove the occupants but also destroy their tents.

When COVID-19 mobility restrictions were in force, some people found shelter in (often overcrowded) apartments and received economic support from their families or through informal networks of acquaintances ( Carillon *et al.*, 2020). Others were utterly helpless. Due to the health emergency, administrative processes and asylum procedures were put on hold, court appointments were postponed, and papers were blocked at local prefectures. Many were so afraid of being caught by the police that they opted to stay home and obtain basic necessities through their networks. They perceived themselves as easy targets. As Carillon *et al.* put it, their conduct was dictated by a ‘fear of facing the outside’ ( 2020: 3). They had no alternative to staying inside, because of the risk of being caught by the police and receiving a fine that they would not be able to pay. Their ‘conditions of precariousness’ did not encourage any of the respondents of Carillon *et al.* to ‘immediately produce strategies for circumventing containment’ ( Carillon *et al.*, 2020: 4). The struggles faced by these groups also find mention in the work of Maria de Jesus *et al.* (2022), who highlight how lockdown restrictions severely impacted the lives of asylum-seekers. They experienced food and financial insecurity, social isolation, loneliness, marginalisation, anxiety both for themselves and their families located far away, as well as worries about their uncertain futures, which may involve forced removals ( de Jesus *et al.*, 2022: 7–10).

The isolation and abandonment experienced by asylum-seekers and *sans-papiers* are also highlighted in *La Voix des Sans-Papiers* bulletin ( 2022), published by the Coordination Sans Papiers 75, generally known as CSP75. I met a few CSP75 members at Place de la République, where they gather on Friday afternoons for their weekly demonstration. Despite the mobility restrictions and ban on public gatherings, CSP75 organised public events on 24th April, 30th May and 20th June 2020. The bulletin highlights the many difficulties that the *sans-papiers* had to face during the lockdown. It also reveals their motives for organising public demonstrations during this period of strict immobility and (forced) confinement. In particular, the two-month-long closure of construction sites was extremely problematic for many *sans-papiers* and irregular workers ( *travailleurs au noir*), who were employed at those sites ( *La Voix des Sans-Papiers*, 2022: 3). They found themselves not only without work but were also ineligible for unemployment support. Those who continued working experienced major difficulties moving around Paris, not just because they had to show *attestations de déplacement dérogatoire*, but also because this certificate had to be shown along with identity documents. *Sans-papiers* were thus ‘doubly exposed’, as Samira ^8^ explained: to getting COVID-19 and to being subjected to control every time they moved. ^9^ To use her own words:

One of the things that touched me about our quotidian life was that in order to move during the pandemic we had to have the *attestations de déplacement dérogatoire*, and together with that authorisation […] we had to show our identity documents. Then, for us there was a double pressure. We were doubly exposed to being controlled as *sans-papiers*. […] I haven’t worked but for the comrades [ *camarades*] who worked during COVID, it was a time of absolute stress. Just imagine the stress because of COVID, the stress because we could not understand what that disease was, and the stress to move around. Those who had the ability to move were very courageous.

During the lockdown, Samira stopped working as a babysitter, and consequently, she lost out on her monthly salary. Moreover, she did not get any support because, at that time, she was *sans-papiers*. ^10^ Her worst fear during the pandemic was contracting the virus and dying in France. As she put it:

I would like to say something very banal: I did not want to die and be buried in France as there were no flights between Tunisia and France. The very idea that I could have died — as anyone could have died during that time, as no one really knew what COVID was — [was terrifying]. Suddenly I said to myself, I don’t want to die because we, our bodies, could not be expatriated, as […] there were no flights.

Among the *sans-papiers*, the worst off were probably those in informal camps, on the outskirts of Paris. Immobility rules negatively impacted their everyday living conditions. Also, the support and assistance that they had received before the pandemic stopped during the lockdown. Especially terrible were the living conditions of unaccompanied minors, whose state of abandonment has been acknowledged by many non-governmental organisations in recent years ( Oberti, 2021).

Despite the restrictions, Lejla ^11^ did not stop providing support to unaccompanied minors. I now consider her narrative.

## ‘Sneaking around like a mouse’

Before the pandemic, Lejla ^12^ was associated with several organisations supporting *sans-papiers*, especially unaccompanied minors, who are overwhelmingly excluded from the official protection system, under the assumption that they fake their ages. ^13^ The only entity involved in supporting unaccompanied minors, at the time, was the Red Cross, which was in charge of DEMIE, ^14^ the unit responsible for assessing the age of unaccompanied minors. ^15^

Given minors’ state of abandonment, Lejla was mostly engaged in delivering blankets, clothes, food and basic necessities to those sleeping in unauthorised camps, comprised of donated tents. During COVID-19, volunteers were unable to go to the camps and the ‘materials and donations stopped flowing’ as well. Before COVID-19, such distribution took place in public spaces—for instance, Gare de Lyon, Place de la République, Stalingrad or Aubervilliers—where both the distributors and the receivers were visible. However, the mobility restrictions complicated assistance and solidarity actions. As Lejla reported:

The most difficult part was to deliver stuff to the camp. The problem was how to reach the other side of Paris during COVID-19. […] They have been forced to move to an industrial zone — Aubervilliers, by the river under the bridge, where nobody could see them, as if they didn’t exist. But the police knew where they were.

During the COVID-19 pandemic, ‘NGOs were decimated’. This had negative repercussions for refugees, who had to go through a difficult process of adjustment. Most of them were used to being forced to move from one area to another. But immobility was an even bigger problem. They could not travel to Paris anymore, and worse, ‘they could not even go to the supermarket or pharmacy on their own’. Because refugees could not move around, Lejla went to them:

So basically, what I did, and many people in Paris did, was to understand how to move through the city. I took lots of risks and I had lots of those bunches of paper, printed out, with me — *déplacement professionnel*, *déplacement privé* or *déplacement* as entrepreneur. I have always managed. I could move because I was working in education. […] There were ways of avoiding all kinds of restrictions […]. The police were so preoccupied with those breaking the law and restrictions that they did not pay attention to the refugees. Police were chasing people on the street, and everyone in cars without authorisation. […] When you got to the camp, they did not even know what COVID was. They had no idea. They just knew that they couldn’t move.

What Lejla highlighted was that, for refugees, COVID-19 ‘was not really *the* issue’. They had to deal with so many other imminent problems. COVID-19 was not perceived as a big problem in their lives, as they were already threatened by the fact that they had to sleep ‘in the street, with rats, in the garbage, with no way of washing themselves’. So, a part of Lejla’s work was to go with them to the pharmacy or to ask what they needed and try to get it for them. When asked whether the situation was challenging for her, she replied with the following:

I was in shock given the entire atmosphere, which reminded me of the beginning of the war in Sarajevo. […] But because at that time I was working in education. […] I kept the authorisations that my head had signed. Hence, I did not suffer that much because of COVID in terms of financial issues. I adapted very quickly, and I started to figure out which papers I needed, where to show them, where to go. I did lots of things that were not allowed. This is what you do. I had a lot of things in my basement. I would grab some bags and I would ask people to help me with the bags. And it worked. Working in education […] with kids, I was authorised to move. I was stopped a few times. […] but never punished. Not even once. I, somehow, fit in. In this area, no one was targeting me for something. So I got by.

When I asked her what other organisations had done during the first months of the pandemic, and why they had not been particularly active, she acknowledged that they had done little, but added that ‘it was much more difficult for them to be active’. As she put it:

I, sneaking around as a mouse, and bringing bags to refugees, and coming back without carrying anything, was fine as I was not that visible, and not connected with any political organisations. […] That’s another thing. Big organisations could do it: Red Cross and Médecins du Monde could do it. But they did very little. Red Cross showed up only once; perhaps they thought it was risky. The only help refugees on the streets got was from grassroots associations, and from the people who *reacted* because they felt compelled given the situations they saw in front of their homes or in the streets of the city.

When the concept of ‘reaction’ came up during the conversation, I asked for clarifications. However, before responding, Lejla made a suggestive comment that touched upon the concept of ‘illegal’, even though during our conversation, the concept of ‘illegality’ had never arisen: ‘I always say that slavery was legal and helping slaves was illegal! [But] I do not care about legal/illegal anymore’. Lejla shared further thoughts on this:

I really wanted to act. I decided what to do, not necessarily following the law. The law is made for certain groups of people, not for everybody. I have the ability to interpret the law. […] COVID was not a personal issue. We were in it together. Being part of a society means taking care of people who are vulnerable, who are old, who are on the streets, who are homeless or refugees. We cannot separate ourselves from them.

## The ability to react

Towards the end of our conversation, and because I was eager to know more about her thoughts on the concept of reaction, I asked whether she thought that people tended to react because they felt a sense of solidarity or, perhaps, a sense of responsibility. She offered the following response:

I think the French people […] have been educated in terms of social responsibility and social rights, and I can see that they are freer now than when I came here. They feel that they have a right to act according to their own judgements, feelings and emotions, and sense of morality, when they see something that is not OK. They do not leave it to the police, the state or whatever. If they see injustice, they act on it. It is not necessarily political engagement. It is not charity. But I think it is still political, in the sense of a desire to correct injustice. Nobody would think: I am saving them. It is more the idea that the government is ‘fucked up’, and they are not doing their job, […] so I force them to make these people visible, to help and force them to do their job.

Lejla’s approach to COVID-19 restrictions was not dissimilar to that adopted by residents of La Goutte d’Or, an area in the north part of Paris that has historically been attentive to the problems of refugees. ^16^ Here, the involvement of many was also grounded in a distinctively political perspective. This was made clear, from the beginning of our conversation, by Louis, a local resident engaged in voluntary work in that area. ^17^ His participation, during the time of COVID-19 immobility restrictions, started about three weeks after the beginning of the lockdown, that is, during *la première phase du confinement*. The initiative, he explained, was taken by an Italian organisation, *Brigades de Solidarité Populaire*, ‘an anarchist, communist, revolutionary Italian group’, of which he was not a member. ^18^ Still, he was contacted because he was a volunteer at the local library. He joined the initiative only after it was clear what they were going to do: prepare food for those out of work.

The food was prepared in a local bar, whose owner allowed volunteers to use the kitchen. As Louis explained, in that neighbourhood, many people worked full-time but without contracts. This meant that during COVID-19, they did not receive a salary. Many local people started ‘meeting and participating’ in the activities of *Brigades de Solidarité Populaire*. Louis ‘started with three people, by going to the market to get some food, and then organised free distribution […] three times a week’. When asked how people were able ‘to meet’, given the mobility restrictions, he clarified that ‘because it was for helping people, we had certificates explaining why we were working in the streets. I wrote down that I was helping poor people’. When asked to clarify who was involved in these activities, he said, ‘political people’ — political in the sense that they had ‘a consciousness in politics, […] from extreme left’ to centre-left.

The starting group of three gradually became a group of some 40 to 50 people. Initially, most people stayed at home, but they began to take part after a few weeks when it was clear that many were in need. Louis himself respected the stay-home policy for three weeks. But he soon realised that *Brigades de Solidarité Populaire* was a good initiative, and ‘decided to come every day […] until the end of the confinement. Once every place reopened, we did not need to continue organising the action’. Indeed, the action was possible because, in that neighbourhood, people knew each other. But political standing, of ‘ *le groupe centrale*’, was a key element in their willingness to react.

When asked his opinion on whether the action was motivated by charity, solidarity or a sense of civic responsibility, Louis stated the following:

*Brigade de Solidarité Populaire* is not a charity. […] We did not want to have this attitude. […] But we tried to make people autonomous. But this is really hard. […] We want to live on a road where everyone takes their own decisions. […] Let’s say that solidarity is very close to charity: they needed our help and we helped. But solidarity, from my perspective, also involves a political standing. We are responsible as citizens, […] *une action politique dans une conscience politique. C’est légitime*. ^19^ […] Those who are committed try to create something new, and try to make it possible. Some collectives are still active. […] But, here, for us, it is over. […] It was a good thing, but as every good thing, it came to an end.

In his concluding remarks, Louis made it clear that, in his neighbourhood, people were sensitive to social issues that concerned their area. About public demonstrations he said, ‘we go together to the demonstrations. We have been doing this since before the pandemic’.

## Which (civic) responsibility?

The narratives so far considered, despite the differing contexts, have a key, and common, element: continuity. Those committed to assisting people in pre-pandemic times did not necessarily change their attitudes in the face of a life-threatening event. The question of how to understand, and even judge, these mobility practices is important, as the acts were performed doing an emergency, when people were asked to conform to certain rules. To what extent are ethically driven mobility practices responsible acts? And if they were deemed responsible acts, against whom were they responsible?

If we look at these practices from a strictly *ethical* perspective—apart from traditional debates on civic virtues, which tend to focus on citizens’ political engagement—they are responsible because ethically driven. Their key aim was to provide support, and the people who got involved believed that the only way to achieve this was to defy the dominant mobility restrictions. Such practices are different from the self-oriented counter-practices that were investigated in Italy ( Puggioni, 2022): those practices aimed primarily at satisfying people’s desire to be out of their homes and to create some semblance of normalcy during an exceptional time. That work suggests, among other approaches, reading counter-practices through the work of Michel de Certeau (1984), which highlights how people utilise, transform, adapt and manipulate systems from within, by operating upon products, procedures, norms and principles. In this article, it seems more pertinent to read these mobility practices through the work of Jacques Derrida, not only because of the ethical implications of responsibility, but more importantly, because responsibility and irresponsibility are inherently inseparable.

But before shifting attention to Derrida’s work, it is important to clarify that Derrida’s ethical approach builds on the work of Emmanuel Levinas (1969), who was the first to highlight the centrality of ethics to the self–Other relationship. Given the influence of Levinas’ ethics in Derrida’s work, I must briefly elucidate on the concept of responsibility according to Levinas.

To begin with, not only did Levinas place the self–Other relationship at the centre of his analysis, but he also moved away from the Cartesian rational and autonomous subject. More specifically, the ethical subject is not the Kantian rational and autonomous being who chooses and calculates the most appropriate ethical action according to universal laws, but a ‘sentient self ( *un soi sentant*)’ ( Critchley, 1999: 239). That is, an affective self, open and sensible towards the Other, with whom the self creates an ethical relationship. As articulated in Joshua Shaw’s analysis, for Levinas, to ‘recognize another person as a person is not to recognize a mind but […] a being who depends on me for help’ ( Shaw, 2008: xxvi). It is the Other’s condition of need and vulnerability that generates a sense of responsibility. This responsibility emerges because the self is not free to (choose to) respond, but it feels obliged to do so. Responsibility, from this perspective, is but the ability ‘to respond to [ *répondre à*]’ the need of the Other ( Raffoul, 2014: 415). These are the premises upon which Derrida developed a distinctive ethics of responsibility, mostly articulated through the concept of the impossible.

Derrida connects responsibility to an impossible event. As he puts it, responsibility is the ‘experience and experiment of the possibility of the impossible’ ( Derrida, 1992: 41). François Raffoul further clarifies that Derrida’s thought is articulated around ‘the impossible as possible and the possible as impossible’ ( 2008: 273). The impossible is not simply treated as the opposite of the possible ‘but, on the contrary, would be what “haunts the possible”, what truly “enables” or possibilizes the possible’ (273). The impossible event is but the unexpected and unthought experience ‘[h]appening outside of prior conditions of possibility (and therefore ‘im-possible’)’ ( Raffoul, 2014: 423).

According to Niall Lucy (2004: 107), Derrida’s concept of responsibility is ‘irreducible either to a programme (a code of ethics, a set of social obligations or political duties) or the opposite of a programme (intuition, solipsism, anarchy)’. It cannot be otherwise, as the ability to respond to an unexpected event emerges outside of established norms, habits and customs. The ability of the self to respond to the unexpected is articulated by Derrida (1995b: 7) using the concept of ‘response-ability’ — an ability that emerges only by moving ‘beyond the very language of *duty*’ imposed by dominant norms. By acting beyond the law, the self engages with the impossible, that is, with a non-calculated, unexpected and unpredictable encounter, which requires a ‘dissident and inventive rupture with respect to tradition, authority, orthodoxy, rule, or doctrine’ ( Derrida, 1995a: 27). The moment of responsibility coincides with the moment of break. As Derrida claims, ‘A decision has to be prepared by reflection and knowledge, but the moment of the decision, and thus the moment of responsibility, supposes a rupture with knowledge, and therefore an opening to the incalculable’ ( Derrida & Ferraris, 2011: 61). This incalculability is not exclusively due to the unexpected event but also, and perhaps more importantly, due to the specific needs of the Other. For Derrida (2007), the ability to respond depends upon the Other, because a decision depends upon, and emerges from, the Other. He clarifies, ‘I’m responsible for the other and it’s for the other that I decide; it is the other who decides in me, without in any way exonerating me from “my” responsibility’ ( Derrida, 2007: 455). But each response results from a difficult choice. Responsibility, from this perspective, expresses a paradox, an aporia, a condition of undecidability. As Raffoul says:

‘Undecidable’ does not mean the impossibility of decision, but its paradoxical condition, that is, its condition of possibility and/or impossibility. […] Decision *remains* confronted with the undecidable that makes it possible *as decision*. A decision must decide without rules to follow, to apply or to conform to, each time a singular decision as an event ( 2014: 423).

The ability to respond *ethically* is thus ‘a matter of *invention*, an invention of the impossible’ ( Raffoul, 2014: 424). It is a creative intervention beyond the external constraints of norms, laws, ways of conduct or habits.

The undecidability is not simply due to an (in)ability to respond to Others’ needs but also to the impossible choice that responsibility demands. More specifically, in *The Gift of Death*, Derrida distinguishes between ‘responsibility *in general* and *absolute* responsibility’ ( 1995a: 61). Citing the biblical story of Abraham who was commanded by God to kill his own son Isaac, Derrida stresses that Abraham was confronted with a double, and equal, responsibility: one towards God and another towards the wider community. Whatever decision he was going to take, his act of responsibility towards one would have generated a correspondent act of irresponsibility towards another. By introducing a distinction between general and absolute responsibility, Derrida highlights not only how the two are interconnected, but also, crucially, the impossibility of any reconciliation between the two. Indeed, absolute response-ability towards the unique Other does not erase a general responsibility towards all others. This is precisely the paradox of undecidability: both events demand responsibility. From this perspective, any decision is always an impossible one: ‘to be responsible to an/the other, one has to be irresponsible to all others’ ( Anderson, 2015: 55). To use Derrida’s own words:

As soon as I enter into a relation with the absolute other, my absolute singularity enters into relation with his on the level of obligation and duty. I am responsible to the other as other, I answer to him and I answer for what I do before him. […] There are also others, […] the innumerable generality of others to whom I should be bound by the same responsibility, a general and universal responsibility […]. I cannot respond to the call, the request, the obligation, or even the love of another without sacrificing the other other, the other others. […] As a result, the concepts of responsibility, of decision, or of duty, are condemned a priori to paradox, scandal, and aporia. Paradox, scandal, and aporia are themselves nothing other than sacrifice ( Derrida, 1995a: 68).

## Concluding remarks

By reading counter-practices through Derrida’s concept of (ir)responsibility, this article highlights the difficulty, if not the impossibility, of judging ethical acts through an either/or approach. What made a good, and thus responsible, citizen during COVID-19 was, and still is, difficult to assess. The mobility restrictions introduced during the pandemic did not simply limit political participation—by curbing protests, public gatherings and collective forms of political engagement—but also restricted people’s ability to respond to the specific needs of particular (marginalised and invisible) groups.

However, the question of what makes a citizen (ir)responsible during a time of emergency is crucial from both a theoretical and practical perspective. Which form(s) of citizenry should prevail during local, national or international emergencies? Does a state of emergency require (exclusively) an obedient subject who conforms, without deviating, to dominant, even if illiberal, norms? If it is the case, does this mean that the liberal, democratic, politically and ethically engaged subject should be unmade during an emergency?

## Ethics and consent

The study was conducted in accordance with the Declaration of Helsinki and approved by the Ethics Committee of Luiss University (Italy), on 21 September 2021, where the project was carried out. Written informed consent was obtained from all subjects involved in the study.

## Data Availability

DANS-EASY: *COVID-19 and (ir)responsible mobility: Reading counter-practices through Derrida*. https://doi.org/10.17026/dans-xkn-ehkp ( Puggioni, 2023b). The project contains the following underlying data: -   All interviews.pdf DANS-EASY: COREQ checklist for ‘COVID-19 and (ir)responsible mobility: Reading counter-practices through Derrida’. https://doi.org/10.17026/SS/YKPF0L ( Puggioni, 2023c). Data are available under the terms of the Creative Commons Attribution 4.0 International license (CC-BY 4.0).

## Footnotes

^1^ In this article, I refer to “asylum-seekers”, “refugees” or *sans-papiers* irrespectively of their effective legal status. I reproduce the definitions that my respondents used.

^2^ The desk work was carried out both in Italy and in Paris. I spent three weeks in the French capital between the end of November and the middle of December 2022. The interviews were open-ended, as the key aim was to allow the stories, narratives, and thoughts of respondents to emerge.

^3^ The qualitative data from the interviews are publicly available ( Puggioni, 2023b).

^4^ Seven questions were made available in a A4 sheet, in French, and revolved around the following: 1) which organisations they were involved with and which activities they regularly carried out; 2) what impact did COVID-19 have on their lives, especially during the first stage of confinement; 3) what made them choose to remain active, if they did, during the pandemic; 4) what protective measures did they resort to; 5) which feelings were dominant, as for instance insecurity or anxiety; 6) how would they describe their (attitudinal) involvement with the organisation; 7) any other comments and thoughts they were willing to share. On average, the interviews lasted between 45–75 minutes.

^5^ The second round of elections was held on 28 June 2020.

^6^ The translation from French to English is mine.

^7^ The concept of ‘the uncounted’ ( Rancière, 1999: 38) is perhaps the most fitting for this context. The ‘uncounted’ are, for Rancière, the invisible, the marginalised, those not counted as part of the community.

^8^ Samira is a fictional name, as are all the other names used in this article. However, in the process of “renaming” the person, I have taken into consideration their gender and nationality. Samira is a Tunisian, who consented to talk to me, on 16 December 2022, at Place de la République, during the Friday weekly gathering. The interview was conducted in French but, while listening to her, I formulated some extra questions in English rather than in French, as they came to me spontaneously, and I was aware that Samira could understand English.

^9^ It goes without saying that the politics of control and surveillance against *sans-papiers*, as well as the criminalisation of any act of solidarity toward them, pre-existed COVID-19 (see Dadusc & Mudu, 2022; Fekete, 2018; Ilker *et al.*, 2017; López-Sala & Barbero, 2019; Puggioni, 2021).

^10^ As of 2022, after six long years in France, her legal status has been regularised.

^11^ Lejla is a volunteer who has been working with Care4Calais since The Jungle was demolished and many unaccompanied minors came to Paris in 2016. I approached her during my fieldwork in Paris through a network of scholars who have been engaged, academically and voluntarily, with *sans-papiers* and asylum-seekers. She is from Sarajevo and became a refugee when she was 14. Our conversation took place on 13 December 2022. It was primarily in English, with only a few words in French.

^12^ Unless otherwise stated, the contents of this section come from my long conversation with Lejla.

^13^ She noted that unaccompanied minors tend to look older than they are. In fact, she looked 40 when she left Sarajevo, when she was only 14.

^14^ DEMIE stands for *dispositif d’evaluation des mineurs isolés étrangers* (assessment system for unaccompanied foreign minors). Since July 2022, France Terre d’Asile has been in charge of the age assessment process.

^15^ Human Rights Watch has been especially critical of Red Cross activities, and the title of its report is telling: Like a Lottery ( 2018).

^16^ La Goutte d’Or is a multi-ethnic and multi-linguistic neighbourhood located in the 18th arrondissement. Traditionally, it has been a working-class area, and since the post-WWII period, one with a high concentration of residents from Maghreb and Sub-Sahara. This area is especially known for Saint-Bernard de la Chapelle Church, which became a sanctuary for some 300 *sans-papiers* in June 1996. Next to the church, the organisation Salle Saint-Bruno is active in the neighbourhood, and particularly sensitive to issues of equality, rights and refugee inclusion. On 15 December 2022, while carrying out my fieldwork in Paris, I attended the meeting organised in light of the forthcoming public event to be held on Sunday, 18 December, on the occasion of International Migrants Day, under the slogan ‘ *Solidarité, liberté, égalité, papiers*’.

^17^ The conversation with Louis took place on 15 December 2022, mostly in English. I approached Louis, a few days before, at a local thrift store, in Rue Léon, where he was doing voluntary work. He agreed to share his experience.

^18^ The Brigades de Solidarité Populaire was still active at the time of my fieldwork, in La Goutte d’Or, mostly through a local library, small shops selling used items and arranging food distribution drives.

^19^ ‘A political action within a political conscience. It is legitimate.’
