# COVID-19 and (ir)responsible (im)mobility: Reading counter-practices through Levinas and Derrida

**DOI:** 10.12688/openreseurope.16686.2

**Published:** 2024-10-07

**Authors:** Raffaela Puggioni

**Affiliations:** 1 Jindal School of International Affairs, OP Jindal Global University, Sonipat, Haryana, 131001, India

**Keywords:** Civic responsibility, lockdown, immobility, sans-papiers, Paris, ethics

## Abstract

The COVID-19 pandemic has affected virtually all daily activities, relations and practices. People were expected to act responsibly by following social distancing, masking, sanitation and stay-home rules. The prevailing ethos of the time was that to protect others, one must first protect oneself. By examining the creative modalities through which (a few) people in Paris circumvented mobility restrictions to help and support those in need, this article investigates the relation between (im)mobility and (ir)responsibility. Is mobility, during a time of forced immobility, an irresponsible act? What does it mean to act responsibly during a life-threatening emergency? Does responsibility always require complete and unequivocal compliance with extant norms, or should responsibility *also* be evaluated in light of the motives that inspire (unauthorised) mobility? The issue of (ir)responsible (im)mobility is scrutinised here by drawing upon the work of Emmanuel Levinas and Jacques Derrida. While the former furthers our understanding of ethical relations, the latter makes us rethink the concept of response-ability and, in particular, the aporia this concept entails. As Derrida highlights, truly ethical acts are impossible for the very reason that all ethical acts are, at the very same time, responsible towards some and irresponsible towards others.

## Introduction

A great deal has been written about the COVID-19 pandemic, given its deadly impact on our daily lives. It disrupted, challenged, altered, slowed down and, at times, suspended usual activities, relations, practices and behaviours. Political scientists have closely investigated its societal impacts, including the following: emergency powers and the state of exception ( Csernatoni, 2020; Ginsburg & Versteeg, 2021; Spadaro, 2020); technologies of control and surveillance ( Bigo *et al.*, 2021; Eck & Hatz, 2020); political participation and democracy ( Afsahi *et al.*, 2020; Borbáth *et al.*, 2021); governmentality and (im)mobility ( Shin, 2021; Wolff *et al.*, 2020); as well as (il)liberal policies and activism ( Bieber, 2022; Pleyers, 2020; Pressman & Choi-Fitzpatrick, 2021).

Mobility restrictions—together with other mandatory protective measures, such as quarantining, social distancing and sanitation practices—were particularly challenging to implement and follow. Due to the imposition of immobility rules, many daily activities were restricted, if not wholly suspended. Worldover, people resisted the restrictions through counter-practices, protests, acts of evasion and civil disobedience. It is precisely these counter-practices that my paper focuses on. Discussing the relation between (im)mobility and (ir)responsibility will contribute to the existing literature by shedding light on a few questions: is mobility—during a time of forced immobility—an irresponsible act? What does it mean to act responsibly during a life-threatening emergency? Does responsibility necessarily entail complete and unequivocal compliance with extant norms? Or should responsibility be *also* evaluated in light of the motives underlying the (unauthorised) mobility? By reflecting on the concept(s) of (ir)responsible (im)mobility, this work complements the existing literature by introducing an ethical perspective to existing works, most of which investigate mobility through the prism of freedom, political activism, democratic participation and resistance ( Celermajer & Nassar, 2020; Degerman *et al.*, 2023; Greitens, 2020; Kriesi & Oana, 2022; Neumayer *et al.*, 2023; Plümper *et al.*, 2021; Zajak *et al.*, 2020).

The numerous public protests in Europe against lockdown measures and/or compulsory vaccination were widely controversial ( Hurford, 2022; Juen *et al.*, 2023; Munir & Munir, 2023). By disregarding dominant norms pertaining to social distancing, mask-wearing and stay-home policies, people were not only jeopardising their own lives but also threatening the well-being of the population at large. In light of this, all unauthorised movements, especially during the lockdown, were considered irresponsible. In their article, Thomas Jacobus de Jong and Carina van de Wetering highlight this and note that the ‘fight against the coronavirus demands from me that I not merely follow the rules for my own sake, but also for the sake of the other’, and that ‘to abide to […] rules is a show of support in the spirit of solidarity’ ( 2021: 151). Seen through the prism of solidarity, respect and good citizenship, abiding by norms was considered not simply a legal concern but an ethical one. During the COVID-19 pandemic, being a good citizen no longer meant being autonomous and liberal—free to decide how to behave—rather, it entailed being a law-abiding individual. Konstantinos Papageorgiou summed it up when suggesting that there existed ‘a moral and a legal duty not to harm others and in some cases at least […] a duty to protect others’ ( 2021: 169). Most people—except those working in the medical and essential services sectors—were expected to perform this duty of protecting others by refraining from physical contact with them. In other words, assistance was to be provided from a distance ( Tomasini, 2021). Neither mobility nor direct contact was considered responsible behaviour.

In this article, I focus specifically on (ir)responsible (im)mobility. This is done by exploring some creative modalities through which (a few) people in Paris circumvented mobility restrictions in order to provide help and support to those in need. Rather than adopting the prism of solidarity, as some have done thus far ( Corrias, 2021; Ignácz & Langenkamp, 2021), I will investigate some mobility practices through the prism of (ir)responsibility. I will do so by drawing upon the work of Emmanuel Levinas and Jacques Derrida. While Levinas helps us further our understanding of ethical relations, Derrida makes us rethink the concept of ‘response-ability’ as well as ethical acts, which are, at the very same time, responsible towards some and irresponsible towards others.

I will develop this argument in five steps. Firstly, I will discuss the debate on surrendering individual and collective responsibility during a life-threatening emergency. Thereafter, I will turn to the works of Levinas and Derrida and discuss what it means for them to act ethically and (ir)responsibly. A brief overview of the mobility restrictions implemented in France during the COVID-19 pandemic will follow. The overview will also contextualise the conditions of confinement and invisibility experienced by asylum-seekers ^1^ and *sans-papiers* during the initial months of the pandemic. Finally, I will discuss some mobility practices adopted in Paris and read them through the prism of (ir)responsibility.

## Emergency and the duty to protect

During the pandemic, health and safety were the dominant rationale in all policy-level decision-making. Given the exponential infection rate, isolation and health-protective measures were deemed to be the key to halt the deadly COVID-19 virus. By staying home, maintaining social distance and wearing a face mask, it was possible to protect oneself, and, at the same time, protect others. While the optimal course of action, in terms of preserving the health of the population, was clear, it was less clear how to implement such radical changes in everyday life. Apart from Sweden, which mainly relied on voluntary compliance ( Larsson, 2022), fines and penalties were introduced in most European countries. However, restrictions and enforcement measures varied across countries. The different approaches as well as the differing opinions of (medical) experts encouraged speculation on which measures were necessary and which could be bypassed.

The debate on the emergency measures mostly followed two (irreconcilable) directions, illustrated here in a simplified way. The first point of view emphasised complete compliance with the emergency rules. To curb the spread of the virus, all residents needed to collaborate, and, in particular, people needed to refrain from acting on personal interests. Arguments that equated the COVID-19 pandemic to a state of war and social distancing to one’s patriotic duty to protect the country—by protecting its people—were advanced constantly. The state of emergency required exceptional behaviour as well as exceptional security measures to guarantee the safety of the population ( Ryan, 2023). In the contrary point of view, emergency measures were seen as illiberal and thus unjustified restrictions that obstructed individual freedom. Under this perspective, everyday counter-practices as well as public protests were deemed legitimate. According to this viewpoint, even in an emergency, liberal democracies could not strip citizens of fundamental rights ( Hertel & Buerger, 2023; Norrlöf, 2020). This perspective was often criticised as being individualistic—oriented towards the self and not the overall well-being of the community. As Papageorgiou pointed out, not only may a democratic state ‘legitimately enforce laws and policies in order to protect its citizens from risks to life and limb’, but citizens also have ‘a moral and a legal duty not to harm others and in some cases at least they have a duty to protect others’ ( 2021: 170). There was an obligation to comply with norms, and given the high risk of contagion, it was ‘unreasonable to act as if the reason for the restrictions did not exist’ (172).

During the pandemic years, people performed countless acts of evasion in almost all European countries. However, evasion practices were not the same: the modalities, as well as motives, were often very different. While some resorted to micro-acts of mobility in order to recreate some normalcy, others resorted to public and static protests against mobility restrictions ( Meers *et al.*, 2023; Puggioni, 2022). Still others, the most controversial, invoked the language of rights—to freedom, movement, protest and choice—and organised mass protests against mobility restrictions and compulsory vaccinations ( Gerbaudo, 2020; Kowalewski, 2021; Russell, 2023).

The general debate on (im)mobility and (ir)responsibility rarely considers whether some counter-practices may be considered responsible when oriented towards the well-being of others. Here, I am not referring to minor adjustments—such as removing one’s mask so that one can be recognised by recipients of care ( de Jong & van de Wetering, 2021: 166)—but to some counter-practices wherein people defied mobility restrictions to reach those in need. It is these practices that this article will investigate by asking the following: are ethically-driven mobility practices irresponsible? By ethically-driven mobility, I refer to practices that involve a violation of immobility rules to offer some help or assistance. I do not refer to the concept of solidarity, even if it has become ‘an ethical buzzword during the 2020 corona pandemic’ ( Häyry, 2022: 256). The ‘corona solidarity’ ( de Jong & van de Wetering, 2021: 151) or the ‘pandemic of solidarity’ ( Broom, 2020), refers to activities and behaviours that were aimed at sharing feelings, establishing closeness, and supporting people in one’s neighbourhood during the pandemic ( Häyry, 2022). These included, for instance, singing together from balconies at specific times; displaying rainbow banners as a sign of hope; clapping hands to show one’s gratitude to medical personnel; buying food for the most vulnerable; providing online support; posting messages to raise the spirits of one’s friends, family, and community or suggesting how to spend time at home. Despite the use of the concept of solidarity, these displays aimed more at communicating a sense of community, unity and support for governments, medical personnel and those working in essential sectors rather than having direct contact with those in need, who were mostly helped from a distance ( Häyry, 2022). The support consisted, thus, of staying home and protecting oneself and others by refraining from any external contact. In other words, immobility was considered responsible.

## Who is the ethical subject?

Zygmunt Bauman’s article, ‘What Prospects of Morality in Times of Uncertainty?’ ( 1998), offers a good starting point for reflecting on ethics in general as well as on ethics during challenging times. By drawing upon two biblical stories—the expulsion of Adam and Eve from the Garden of Eden and Moses receiving the Ten Commandments on Mount Sinai—Bauman reflects on the differing moralities that each story suggests. As he put it:

The first story suggests that to be moral is to face a choice between good and evil, and to know that there is such a choice, and make choices with that knowledge. The second story implies that to be moral is to follow strictly the command – to obey unconditionally and never to deviate from the straight path, in deed or in thought ( 1998: 13).

What Bauman finds problematic is not only that most theories of ethical philosophy suggest that to live a moral life one should follow rules and social practices, but also that people prefer this modality. As he puts it, people prefer ‘to be frightened and forced to be moral’, rather than spending a life ‘in the agony of interminable uncertainty’ ( 1998: 13). However, even if strict obedience is preferred ‘in the name of conformity and against dissent’ (14), the encounter with the other does complicate the picture. Rules might not be followed uncritically: choices must be made. At this point, Bauman draws upon the work of Levinas to argue that it is the other, and not the self, which makes ethical relations possible. Starting from Levinas’ claim that ethics is prior to ontology, Bauman highlights that ‘[c]odes and norms are not the *beginning*, but the *end* of moral relationship’ ( 1998: 16). In other words, the encounter with the other does alter one’s priorities radically. It is not obedience but choice that takes precedence when others require help or assistance. But does this apply *also* to life-threatening emergencies? Does the other also take precedence during times of crisis? In other words, what is it that makes a subject an ethical subject? Does the ethical subject *also* act responsibly? If so, then responsible to whom? The works of Levinas and Derrida offer important insights into these questions, and it is to their work that I will now turn.

Levinas’ ethical perspective is unique in the way he conceptualises one’s relation with the other. It is not rationality but an ‘infinite responsibility’ toward the other that shapes human action ( 1969: 244). Not only did Levinas place the self–Other relationship at the centre of his analysis, but he also moved away from the Cartesian conceptualisation of the rational and autonomous subject. More specifically, the ethical subject is not the Kantian rational and autonomous being—who chooses and calculates the most appropriate ethical action according to universal laws—but the ‘sentient self ( *un soi sentant*)’ ( Critchley, 1999: 239), that is, an affective self, open and sensible towards the needs of the Other, an Other who invites the self to a face-to-face relation. As Levinas put it, in *Totality and Infinity* ( 1969): ‘The face is a living presence; it is expression. […] The face speaks; […] the face speaks to me and thereby invites me to a relation’ (66, 198). As Joshua Shaw also highlighted, the face-to-face encounter involves not only spontaneous acts of giving, but also an other, who is not a rational thinking subject. As he puts it: ‘To recognize another person as a person is not to recognize a mind but […] a being who depends on me for help. […] [O]ne recognizes oneself as unconditionally responsible for the other’ ( Shaw, 2008: xxvi). By referring to unconditional responsibility, Levinas suggests that the relationship between the self and the other is not dependent on free choice. It is not freedom but ‘individual response-ability’ ( Burvill, 2008, 234) that shapes the encounter between the self and the other. Not only does the self respond to the other, but in responding, the self ‘give[s] to the other even the bread out of one’s own mouth and the coat from one’s shoulders’ ( Levinas, 1991: 55). More specifically, it is the need and vulnerability of the other that generates a sense of responsibility in the self and makes them respond to the call of help. From this perspective, responsibility is nothing but the ability ‘to respond to [ *répondre à*]’ the needs of the Other ( Raffoul, 2014: 415).

By drawing upon Levinas’ ethics, Derrida developed a distinctive ethics of responsibility. While Levinas’ work highlights what makes a self an ethically responsible self, Derrida’s work develops the concept of (ir)responsibility further, postulating that a pure and true ethical subject—and thus an ethical act—is an impossible event. As he puts it, responsibility is the ‘experience and experiment of the possibility of the impossible’ ( Derrida, 1992: 41). As François Raffoul clarifies, Derrida’s thought is articulated around ‘the impossible as possible and the possible as impossible’ ( 2008: 273). The impossible event is the unexpected and unthought experience ‘[h]appening outside of prior conditions of possibility (and therefore ‘im-possible’)’ ( Raffoul, 2014: 423).

According to Niall Lucy, Derrida’s concept of responsibility is ‘irreducible either to a programme (a code of ethics, a set of social obligations or political duties) or the opposite of a programme (intuition, solipsism, anarchy)’ ( 2004: 107). It cannot be otherwise, as the ability to respond to an unexpected event emerges outside of established norms, habits and customs. Derrida (1995b: 7) theorises the ability of the self to respond to the unexpected using the concept of ‘response-ability’, an ability that emerges only by moving ‘beyond the very language of *duty*’ imposed by dominant norms (ibid). By acting beyond the law, the self engages with the impossible, that is, with a non-calculated, unexpected and unpredictable encounter, which requires a ‘dissident and inventive rupture with respect to tradition, authority, orthodoxy, rule, or doctrine’ ( Derrida, 1995a: 27).

The moment of responsibility coincides with the moment of break. As Derrida claims, a ‘decision has to be prepared by reflection and knowledge, but the moment of the decision, and thus the moment of responsibility, supposes a rupture with knowledge, and therefore an opening to the incalculable’ ( Derrida & Ferraris, 2011: 61). This incalculability arises not so much because of the unexpected encounter with the other but because of the other’s specific needs. For Derrida (2007), the self’s ability to respond depends upon the Other because the decision depends upon, and emerges from, the Other. As he clarifies, ‘I’m responsible for the other and it’s for the other that I decide; it is the other who decides in me, without in any way exonerating me from “my” responsibility’ ( Derrida, 2007: 455).

But each such response is the result of a difficult choice. From this perspective, responsibility entails a paradox, an aporia, a condition of undecidability. As Raffoul says: ‘“Undecidable” does not mean the impossibility of decision, but its paradoxical condition, that is its condition of possibility and/or impossibility’ ( 2014: 423). For Derrida, the ability to respond *ethically* is ‘a matter of *invention*’ ( Raffoul, 2014: 424), a creative intervention beyond the external constraints of norms, laws, ways of conduct or habits. This undecidability is not simply due to an (in)ability to respond to Others’ needs but also to the impossible choice that responsibility demands. More specifically, in *The Gift of Death*, Derrida distinguishes between ‘responsibility *in general* and *absolute* responsibility’ ( 1995a: 61). Citing the biblical story of Abraham, who was commanded by God to kill his own son Isaac, Derrida discusses the impossible choice that Abraham faced. Abraham was confronted with a double, and equal, responsibility: one towards God and another towards the wider community. Whatever decision he would have taken, his act of responsibility towards one would have entailed a corresponding act of irresponsibility towards all the others. By differentiating general and absolute responsibility, Derrida highlights not only how the two are interconnected, but also, more crucially, the impossibility of any reconciliation between the two. Indeed, absolute response-ability towards the unique other does not erase a general responsibility towards all others. This is precisely the paradox of undecidability: both events demand responsibility. From this perspective, any decision is always an impossible one: ‘to be responsible to an/the other, one has to be irresponsible to all others’ ( Anderson, 2015: 55). To use Derrida’s words:

As soon as I enter into a relation with the absolute other, my absolute singularity enters into relation with his on the level of obligation and duty. I am responsible to the other as other, […] I answer for what I do before him. […] There are also others, […] the innumerable generality of others to whom I should be bound by the same responsibility, a general and universal responsibility […]. I cannot respond to the call, the request, the obligation, or even the love of another without sacrificing the other other, the other others ( 1995a: 68).

By highlighting the paradox inherent in the concept of responsibility, as much as in the concept of duty, Derrida highlights that ethical acts are *de facto* impossible. The very act of choosing to respond to some, and not to others, transforms that act into an unethical one. Derek Attridge emphasises this clearly when he refers to the ‘impossibility of ethics’ ( 2010: 56). In other words, ethics becomes an impossible event because

if ethics is the simultaneous responsibility toward every other, *as singular other*, and if, as Derrida insists, *tout autre est tout autre*, “every other is wholly other”, ethical behaviour is, from the very first moment […] utterly impossible. […] And if the act of doing justice is always also the act of doing an injustice, ethical acts – acts which involve no injustice – cannot happen ( Attridge, 2010: 59, 63).

What is impossible is not the ability to respond to the needs of the other but to identify a truly ethical, responsible act. Any choice, which selects some and excludes others, is the result of a sacrifice and, thus, an unethical decision. The problem is not that a selection is made, but that, as Derrida writes, ‘I can never justify the fact that I prefer or sacrifice anyone (any other) to the other’ ( 1995a: 70).

In conclusion, what the works of Levinas and Derrida highlight is that ethical relations are always driven by the other in the sense that it is the needs of the other that stimulate action, an action that requires courage and inventiveness. It is not about following orders, norms, and dominant practices but about breaking away from them. However, while it is clear who the ethical subject is, it is less clear, as Derrida’s work suggests, who the (ir)responsible subject is. The few stories narrated in the following sections make us reflect on this aporetic nature of responsibility.

## Methods

Before shifting attention to the research method used during the fieldwork in Paris, a few words on the overall aim of the MOBILISE project—upon which this article is based— are needed. The *Leitmotiv* of the project was to investigate the relationship between emergency, (im)mobility and the liberal subject. The research did not aim to focus on the restrictions *per se*, but on the subjectivities that emerged out of the many practices of evasion, non-conformity and resistance. In this specific work, my ultimate goal was to encourage a debate that looked at the ethics of (im)mobility practices. This is why, when conducting fieldwork in Paris, I focused not on gathering data on the pervasiveness of mobility practices but on exploring which subjectivities emerged from those practices.

Although the original research plan was to investigate Black Lives Matter protests, once in Paris, I re-evaluated my research trajectory and decided to devote greater attention to the experiences of asylum-seekers and *sans-papiers*, that is, the most marginalised and invisible group throughout the pandemic.

During my desk work, ^2^ I collected information on COVID-19 restrictions in France by accessing official reports, interviews, and legal documents available at the National Assembly, Ministry of the Interior, Ministry of Solidarity and Health, and Police Department ( *Préfecture de police*). I investigated the living conditions of the *sans-papier* in Paris by searching both academic articles and newspaper articles. This was done using both Google Scholar and Google search. Special attention was given to the news published in the followings: *Liberation*, *France24*, *Le Monde* and *L’Humanité*. To identify the sources, I used the following keywords, or a combination of them: *sans-papiers* in Paris, COVID-19, public gathering, public protests, acts of solidarity, responsibility, civism, camps, confinement, police controls, and Coordination Sans Papiers 75.

In terms of the research framework, I adopted a grounded theory approach, although I conducted only three interviews. ^3^ It was especially the narratives of my respondents that made me shift my perspective from public and visible events to ethically driven counter-practices. This was possible because my respondents answered my few questions, ^4^ and also shared *their* (embodied) experience of the pandemic. In particular, the narratives of two of them made me rethink counter-practices not in terms of “liberal subjects” ^5^ but through the lens of the (ir)responsible subject.

A final methodological note is necessary. Given the difficulties associated with uncovering everyday ethical practices, which mostly go unnoticed and unrecorded, and the limited number of interviews conducted, ^6^ I recognise that making any generalisations based on this research is problematic. However, my main objective was not to assess how widespread these practices were but to use these narratives as a starting point to reflect—and encourage a broader debate—on ethically-driven mobility during a time of forced immobility.

## COVID-19 restrictions in France: an overview

In France, on 23rd February 2020, phase one of the COVID-19 containment began. On the 29th, public gatherings of more than 5000 people were banned; this figure was reduced to 1000 on 9th March. A few days after, on the 12 ^th^, President Emmanuel Macron announced the closure of all nurseries, schools and universities, and non-essential companies were asked to conduct their work using online platforms ( APUR, 2021: 6). Notwithstanding the surging health crisis, the first round of municipal elections was held in 35,000 municipalities on 15th March. ^7^ Two days after, on the 17 ^th^, at noon, lockdown measures were announced, and a state of health emergency ( *l*’ *état d’urgence sanitaire*) was officially declared ( Assemblée Nationale, 2020).

The first restrictive measures, initially introduced for 15 days, were prolonged—as was the case in other EU countries—until 10th May, which marked the beginning of phase one of the lifting of restrictions. During the 55 days of lockdown, as the L’Humanité (2020) puts it: ‘the whole of France lived under a bell’. ^8^ As in most EU countries, the end of lockdown measures simply meant the relaxation of the most restrictive norms, not the end of the health emergency. For instance, in the 24 hours that preceded the relaxing of lockdown measures, on 11th May, 70 people were reported to have died due to COVID-19. However, the situation was not the same everywhere in France. As Prime Minister Edouard Philippe clarified during a press conference on 7th May, the process of *déconfinement* was the first step in fighting the epidemic, which was to last a few weeks, until 2nd June. Greater relaxation was permitted in the ‘green’ zones, which reported a lower number of infections and hospitalisations, as compared to the ‘red’ zones—for instance, Île-de-France. However, the prime minister still recommended ‘to keep observing, on a voluntary basis, very strict rules of caution’ ( Vie Publique, 2020). As in other EU countries, mask-wearing on public transportation and in shops was made compulsory, as was social distancing. The ban on public gatherings was retained. Between March and May, constant adjustments were made to the restrictions depending on the epidemiological curve (see APUR, 2021: 6–7).

During the lockdown, mobility was permitted only in certain circumstances, and people had to always carry with them a self-certificate—the so-called *attestations de déplacement dérogatoire—*declaring their intended destination and reason for being out. People could leave their homes only if they worked in essential sectors—such as transportation, education, health or the food industry—or needed to buy basic necessities or engage in *individual* physical activities. Those caught outside without proper justification could be subjected to a fine. This was initially set at EUR 35 and was subsequently increased to EUR 135 on 18th March but could also go up to EUR 375 ( France24, 2020a). On the first day of the lockdown, dissuasive mechanisms were put in place: around 100,000 gendarmes and police were deployed to enforce the lockdown measures, and about 4,095 people were found violating the new rules ( France24, 2020b).

According to Jérémie Gauthier, the controls were especially harsh in working-class neighbourhoods ( *quartiers populaires*) with a high concentration of migrant population, hat were ‘under police pressure’ ( 2020: 57). Indeed, the controls reinforced ‘discriminatory and racist treatments’ (57). Gauthier was especially critical of the Ministry of the Interior’s approach and the ways in which controls were implemented. In light of the official figures—provided by Minister of the Interior, Christophe Castaner—Gauthier pointed out that fines were disproportionately imposed on people living in poor areas, which also registered higher infection rates than other areas ( 2020: 59–60). Didier Fassin was also critical of the *inégalité* perpetuated during the COVID-19 pandemic in France. In his article, *L’inégalité des vies en temps d’épidémie* ( 2020), published in the left-wing newspaper *Libération*, he highlighted in particular the poor living conditions inside prisons and the high risk of contagion in tight and overcrowded spaces.

Mobility restrictions—generally referred to as *mesures barrières*—and social distancing requirements remained in place until the end of August 2020. As specified in the general norms that the Ministry of Solidarity and Health ( Ministère des solidarités et de la santé, 2020) issued on 1st June, one-metre physical distancing was to be maintained ‘in all circumstance[s]’ (art. 1.I); public gatherings, reunions or any other activity taking place in a public venue, with more than 10 people present, were ‘prohibited throughout the territory of the Republic’ (art. 3.I). Article 3.V further stated that public events with more than 5000 people were banned until the end of August, and that the local prefect (the local security authority) was in charge of authorising public events depending on the prevailing local conditions and possible risks (art. 3.IV).

In virtually all countries, some groups of people were more vulnerable than others, and France was certainly not an exception. For instance, in both the USA and the UK, minority groups—especially Black and Asian people—experienced higher contagion and mortality rates than the general population and accounted for the largest share among those working in essential capacities ( Laurencin & McClinton, 2020). Although (forced) confinement was challenging for citizens worldwide, some groups were exposed to greater deprivation, restrictions, job insecurity or loss, deterioration of their economic condition and risk of contracting the virus ( Beaman, 2020; Crouzet *et al.*, 2022; Gaudron *et al.*, 2022). Among the disadvantaged, asylum-seekers and *sans-papiers* were probably the worst off. The following sections shift the focus to their experience.

## The ‘uncounted’ ^9^

During the COVID-19 pandemic, the living conditions of asylum-seekers and *sans-papiers* in Paris were extremely precarious (as is still the case), and the majority of unaccompanied minors were abandoned. As soon as ‘The Jungle’—the unauthorised camp in Calais—was closed down in 2016, Paris became the new destination. According to Madeleine Byrne (2021), approximately 3,000 asylum-seekers, refugees and *sans-papiers* moved into informal camps in the city’s northern areas. In recent years, these people have made themselves visible by pitching tents in key Parisian squares or outside well-known buildings, such as Place de la République, Panthéon, Charles-de-Gaulle Airport, France’s Court of Cassation and Place Saint-Gervais ( Taskin, 2022). Such acts of visibility do not normally last long as the police forcibly remove the occupants and destroy their tents.

When COVID-19 mobility restrictions were in force, some people found shelter in (often overcrowded) apartments and received economic support from their families or through informal networks of acquaintances ( Carillon *et al.*, 2020). Others were utterly helpless. Due to the health emergency, administrative processes and asylum procedures were put on hold, court appointments were postponed, and papers were blocked at local prefectures. Many were so afraid of being caught by the police that they opted to stay home and obtain basic necessities through their networks. They perceived themselves as easy targets. As Carillon *et al.* put it, their conduct was dictated by a ‘fear of facing the outside’ ( 2020: 3). They had no alternative to staying inside, because of the risk of being caught by the police and receiving a fine that they would not be able to pay. Their ‘conditions of precariousness’ did not encourage any of the respondents of Carillon *et al.* to ‘immediately produce strategies for circumventing containment’ ( Carillon *et al.*, 2020: 4). The struggles faced by these groups also find mention in the work of Maria de Jesus *et al.* (2022), who highlight how lockdown restrictions severely impacted the lives of asylum-seekers. They experienced food and financial insecurity, social isolation, loneliness, marginalisation, anxiety both for themselves and their families located far away, as well as worries about their uncertain futures, which may involve forced removals ( de Jesus *et al.*, 2022: 7–10).

The isolation and abandonment experienced by asylum-seekers and *sans-papiers* are also highlighted in *La Voix des Sans-Papiers* bulletin ( 2022), published by the Coordination Sans Papiers 75, generally known as CSP75, which brings together a few independent Parisienne collectives of *sans-papiers*. I met a few CSP75 members at Place de la République, where they gather on Friday afternoons for their weekly demonstration. Despite the mobility restrictions and ban on public gatherings, CSP75 organised public events on 24th April, 30th May and 20th June 2020. The bulletin highlighted the many difficulties that the *sans-papiers* faced during the lockdown. It also clarified their motives for organising public demonstrations during this period of strict immobility and (forced) confinement. In particular, the two-month-long closure of construction sites was extremely problematic for many *sans-papiers* and irregular workers ( *travailleurs au noir*), who were employed at those sites ( La Voix des Sans-Papiers, 2022: 3). They found themselves not only without work but were also ineligible for unemployment support. Those who continued working experienced major difficulties moving around Paris, not just because they had to show *attestations de déplacement dérogatoire*, but also because this certificate had to be shown along with identity documents. *Sans-papiers* were thus ‘doubly exposed’, as Samira ^10^ explained: to getting COVID-19 and to being subjected to control every time they moved. ^11^ To use her own words:

One of the things that touched me about our quotidian life was that in order to move during the pandemic we had to have the *attestations de déplacement dérogatoire*, and together with that authorisation […] we had to show our identity documents. Then, for us there was a double pressure. We were doubly exposed to being controlled as *sans-papiers*. […] I haven’t worked but for the comrades [ *camarades*] who worked during COVID, it was a time of absolute stress. Just imagine the stress because of COVID, the stress because we could not understand what that disease was, and the stress to move around. Those who had the ability to move were very courageous.

During the lockdown, Samira stopped working as a babysitter, and consequently, she lost a source of steady income. Moreover, she did not get any state support because, at that time, she was *sans-papiers*. ^12^ Her worst fear, during the pandemic, was contracting the virus and dying in France. As she put it:

I would like to say something very banal: I did not want to die and be buried in France as there were no flights between Tunisia and France. The very idea that I could have died — as anyone could have died during that time, as no one really knew what COVID was — [was terrifying]. Suddenly I said to myself, I don’t want to die because we, our bodies, could not be expatriated, as […] there were no flights.

Among the *sans-papiers*, the worst off were probably those in informal camps, on the outskirts of Paris. Immobility rules negatively impacted their everyday living conditions. Further, the support and assistance that they had received before the pandemic stopped during the lockdown. The living conditions of unaccompanied minors—whose state of abandonment has been acknowledged by many non-governmental organisations in recent years—were deplorable ( Oberti, 2021). Despite the restrictions, Lejla ^13^ did not stop providing support to unaccompanied minors. I now relay her narrative.

## ‘Sneaking around like a mouse’

Before the pandemic, Lejla ^14^ had worked with several organisations supporting *sans-papiers*, especially unaccompanied minors, who are overwhelmingly excluded from the official protection system, under the assumption that they fake their ages. ^15^ The only entity involved in supporting unaccompanied minors, at the time, was the Red Cross, which was in charge of DEMIE, ^16^ the unit responsible for assessing the age of unaccompanied minors. ^17^

Given minors’ state of abandonment, Lejla was mostly engaged in delivering blankets, clothes, food and basic necessities to those sleeping in unauthorised camps, comprised of donated tents. During COVID-19, volunteers were unable to go to the camps and the ‘materials and donations stopped flowing’ as well. Before COVID-19, such distribution took place in public spaces—for instance, Gare de Lyon, Place de la République, Stalingrad or Aubervilliers—where both the distributors and the receivers were visible. However, the mobility restrictions complicated assistance and solidarity actions. As Lejla reported:

The most difficult part was to deliver stuff to the camp. The problem was how to reach the other side of Paris during COVID-19. […] They have been forced to move to an industrial zone — Aubervilliers, by the river under the bridge, where nobody could see them, as if they didn’t exist. But the police knew where they were.

During the COVID-19 pandemic, ‘NGOs were decimated’. This had negative repercussions for refugees, who had to go through a difficult process of adjustment. Most of them were used to being forced to move from one area to another. But immobility was an even bigger problem. They could not travel to Paris anymore, and worse, ‘they could not even go to the supermarket or pharmacy on their own’. Because refugees could not move around, Lejla went to them:

So basically, what I did, and many people in Paris did, was to understand how to move through the city. I took lots of risks and I had lots of those bunches of paper, printed out, with me — *déplacement professionnel*, *déplacement privé* or *déplacement* as entrepreneur. I have always managed. I could move because I was working in education. […] There were ways of avoiding all kinds of restrictions […]. The police were so preoccupied with those breaking the law and restrictions that they did not pay attention to the refugees. Police were chasing people on the street, and everyone in cars without authorisation. […] When you got to the camp, they did not even know what COVID was. They had no idea. They just knew that they couldn’t move.

What Lejla highlighted was that, for refugees, COVID-19 ‘was not really *the* issue’. They had to deal with so many other imminent problems. COVID-19 was not perceived as a big problem in their lives, as they were already threatened by the fact that they had to sleep ‘in the street, with rats, in the garbage, with no way of washing themselves’. So, a part of Lejla’s work was to go with them to the pharmacy or to ask what they needed and try to get it for them. When asked whether the situation was challenging for her, she replied with the following:

I was in shock given the entire atmosphere, which reminded me of the beginning of the war in Sarajevo. […] But because at that time I was working in education. […] I kept the authorisations that my head had signed. Hence, I did not suffer that much because of COVID in terms of financial issues. I adapted very quickly, and I started to figure out which papers I needed, where to show them, where to go. I did lots of things that were not allowed. This is what you do. I had a lot of things in my basement. I would grab some bags and I would ask people to help me with the bags. And it worked. Working in education […] with kids, I was authorised to move. I was stopped a few times. […] but never punished. Not even once. I, somehow, fit in. In this area, no one was targeting me for something. So I got by.

When I asked her what other organisations had done during the first months of the pandemic, and why they had not been particularly active, she acknowledged that they had done little, but she also clarified that ‘it was much more difficult for them to be active’. As she put it:

I, sneaking around as a mouse, and bringing bags to refugees, and coming back without carrying anything, was fine as I was not that visible, and not connected with any political organisations. […] That’s another thing. Big organisations could do it: Red Cross and Médecins du Monde could do it. But they did very little. Red Cross showed up only once; perhaps they thought it was risky. The only help refugees on the streets got was from grassroots associations, and from the people who *reacted* because they felt compelled given the situations they saw in front of their homes or in the streets of the city.

When the concept of ‘reaction’ came up during the conversation, I asked for clarifications. However, before responding, Lejla made a suggestive comment that touched upon the concept of ‘illegal’, even though during our conversation, the concept of ‘illegality’ had not arisen: ‘I always say that slavery was legal and helping slaves was illegal! [But] I do not care about legal/illegal anymore’. Lejla shared further thoughts on this:

I really wanted to act. I decided what to do, not necessarily following the law. The law is made for certain groups of people, not for everybody. I have the ability to interpret the law. […] COVID was not a personal issue. We were in it together. Being part of a society means taking care of people who are vulnerable, who are old, who are on the streets, who are homeless or refugees. We cannot separate ourselves from them.

## The ability to react

Towards the end of our conversation, and because I was eager to know more about her thoughts on the concept of reaction, I asked whether she thought that people tended to react because they felt a sense of solidarity or, perhaps, a sense of responsibility. She offered the following response:

I think the French people […] have been educated in terms of social responsibility and social rights, and I can see that they are freer now than when I came here. They feel that they have a right to act according to their own judgements, feelings and emotions, and sense of morality, when they see something that is not OK. They do not leave it to the police, the state or whatever. If they see injustice, they act on it. It is not necessarily political engagement. It is not charity. But I think it is still political, in the sense of a desire to correct injustice. Nobody would think: I am saving them. It is more the idea that the government is ‘fucked up’, and they are not doing their job, […] so I force them to make these people visible, to help and force them to do their job.

Lejla’s approach to COVID-19 restrictions was not dissimilar to that adopted by residents of La Goutte d’Or, an area in the north part of Paris that has historically been attentive to the problems of refugees. ^18^ Here, the involvement of many was also grounded in a distinctively political perspective. This was made clear, from the beginning of our conversation, by Louis, a local resident engaged in voluntary work in that area. ^19^ His participation, during the time of COVID-19 immobility restrictions, started about three weeks after the beginning of the lockdown, that is, during *la première phase du confinement*. The initiative, he explained, was taken by an Italian organisation, *Brigades de Solidarité Populaire*, ‘an anarchist, communist, revolutionary Italian group’, of which he was not a member. ^20^ Still, he was contacted because he was a volunteer at the local library. He joined the initiative only after it was clear what they were going to do: prepare food for those out of work.

The food was prepared in a local bar, whose owner allowed volunteers to use the kitchen. As Louis explained, in that neighbourhood, many people worked full-time but without contracts. This meant that during COVID-19, they did not receive any salary. Many local people started ‘meeting and participating’ in the activities of *Brigades de Solidarité Populaire*. Louis ‘started with three people, by going to the market to get some food, and then organised free distribution […] three times a week’. When asked how people were able ‘to meet’, given the mobility restrictions, he clarified that ‘because it was for helping people, we had certificates explaining why we were working in the streets. I wrote down that I was helping poor people’. When asked to clarify who was involved in these activities, he said, ‘political people’ — political in the sense that they had ‘a consciousness in politics, […] from extreme left’ to centre-left.

The starting group of three gradually became a group of some 40 to 50 people. Initially, most people stayed at home, but they began to take part after a few weeks when it was clear that many were in need. Louis himself respected the stay-home policy for three weeks. But he soon realised that *Brigades de Solidarité Populaire* was a good initiative, and ‘decided to come every day […] until the end of the confinement. Once every place reopened, we did not need to continue organising the action’. Indeed, the action was possible because, in that neighbourhood, people knew each other. But political standing, of ‘ *le groupe centrale*’, was a key element in their willingness to react. When asked his opinion on whether the action was motivated by charity, solidarity or a sense of civic responsibility, Louis stated the following:

*Brigade de Solidarité Populaire* is not a charity. […] We did not want to have this attitude. […] But we tried to make people autonomous. But this is really hard. […] We want to live on a road where everyone takes their own decisions. […] Let’s say that solidarity is very close to charity: they needed our help and we helped. But solidarity, from my perspective, also involves a political standing. We are responsible as citizens, […] *une action politique dans une conscience politique. C’est légitime*. ^21^ […] Those who are committed try to create something new, and try to make it possible. Some collectives are still active. […] But, here, for us, it is over. […] It was a good thing, but as every good thing, it came to an end.

In his concluding remarks, Louis emphasided that, in his neighbourhood, people were sensitive to social issues that concerned their area. About public demonstrations he said, ‘we go together to the demonstrations. We have been doing this since before the pandemic’.

## Concluding remarks

The COVID-19 pandemic was an extremely challenging experience for everyone. People were requested to radically change their behaviour by restricting their freedom: freedom to move, work, choose, meet people and have a social life. However, mobility restrictions did not make people stand still. Counter-practices, protests, acts of evasion and civil disobedience emerged. The question of whether those practices should be deemed responsible is contested. As contested is the relation between (im)mobility and (ir)responsibility because the dividing line between responsible and irresponsible behaviour does not run simply between conformity and non-conformity. The stories I have recalled here highlight that the encounter— whether direct or indirect—with those in need instils a change of perspective. It makes people decide where they (choose to) stand. To act responsibly, as both Levinas’ and Derrida’s works suggest, individuals need to make a choice—a choice that originates from a personal motive and endeavour and not a collective one. The stories of Samira, Lejla and Louis all suggest that, despite the COVID-19 restrictions, choices were not only unavoidable but also required inventiveness and creativity. All the mobility acts I recorded through their stories—finding ways of ‘sneaking around like a mouse’, moving around the city, buying medicines and basic necessities, making food for those without jobs at a local bar, etc.—highlight that their decision to do something, to react, did not arise from a self-oriented attitude but from the very specific needs of the others, which they did not want to ignore.

However, their acts did not occur in isolation. Lejla mentioned that many locals had volunteered to help those in need. Louis referred to an initial team of three people that grew to 50. Lejla was the one, more than others, who demonstrated that the line between conformity and non-conformity, between legal and illegal, is not as straightforward when acting for the benefit of others. For her, being responsible meant deciding what to do rather than just obeying rules. According to her, laws should not be obeyed passively, instead, they should be interpreted.

Moreover, all three of them acted not in solidarity—as none of them used that specific concept—but out of a sense of responsibility. Still, the responsibility to which they referred was not civic responsibility—that is, being a good citizen who conforms to laws and norms without questioning them. Instead, their choices reflected that of an ethical one. Their acts were driven by a desire to do something good for others, whom they knew were in need. Choosing to help others by taking personal risks also suggests that the (ethical) subjectivities that emerged were oriented towards the other and not towards the self. In other words, by prioritising the other’s needs over their own safety, they challenged the key assumption of the pandemic: that the fight against the coronavirus pandemic required caring for and protecting, first and foremost, oneself, and, by doing so, the protection of others was also guaranteed.

To conclude, the issue—who was the (ir)responsible subject during the pandemic—is no doubt contentious. What the findings of this article suggest is that *civic* responsibility—that is, the duty to respect rules and norms—is not the only responsibility to be considered. The *personal* response-ability to respond to the needs of the Other is equally important. ^22^

## Ethics and consent

The study was conducted in accordance with the Declaration of Helsinki and approved by the Ethics Committee of Luiss University (Italy), on 21 September 2021, where the project was carried out. Written informed consent was obtained from all subjects involved in the study.

## Data Availability

DANS-EASY: *COVID-19 and (ir)responsible mobility: Reading counter-practices through Derrida*. https://doi.org/10.17026/dans-xkn-ehkp ( Puggioni, 2023a). The project contains the following underlying data: All interviews.pdf DANS-EASY: COREQ checklist for ‘COVID-19 and (ir)responsible mobility: Reading counter-practices through Derrida’. https://doi.org/10.17026/SS/YKPF0L ( Puggioni, 2023b). Data are available under the terms of the Creative Commons Attribution 4.0 International license (CC-BY 4.0).

## Footnotes

^1^ In this article, I refer to “asylum-seekers”, “refugees” or *sans-papiers* irrespectively of their effective legal status. I reproduce the definitions that my respondents used.

^2^ The desk work was carried out both in Italy and in Paris. I spent three weeks in the French capital between the end of November and the middle of December 2022. The interviews were open-ended, as the key aim was to allow the stories, narratives, and thoughts of respondents to emerge.

^3^ The qualitative data from the interviews are publicly available ( Puggioni, 2023a).

^4^ Seven questions were made available in a A4 sheet, in French, and revolved around the following: 1) which organisations they were involved with and which activities they regularly carried out; 2) what impact did COVID-19 have on their lives, especially during the first stage of confinement; 3) what made them choose to remain active, if they did, during the pandemic; 4) what protective measures did they resort to; 5) which feelings were dominant, as for instance insecurity or anxiety; 6) how would they describe their (attitudinal) involvement with the organisation; 7) any other comments and thoughts they were willing to share. On average, the interviews lasted between 45–75 minutes.

^5^ By “liberal subjects”, I refer to the liberal attitude that people continued to adopt despite (severe) restrictions. As discussed in my previous work ( Puggioni, 2022), I assume that European citizens—irrespective of the restrictions enforced on them in the attempt to contain the virus—did not abandon their liberal approach to life. In other words, despite the pandemic, European citizens did not transform themselves into docile and obedient bodies ( Foucault, 1995) at the mercy of governmental (illiberal) decisions—nor did EU governments intend to effect such a change.

^6^ Strictly speaking, the respondents were not chosen; rather, I came across them. Nicolas Jaoul was my starting point in illustrating what was happening in the La Goutte d’Or neighbourhood. He shared some of his contacts with me. While Lejla was contacted through an informal network, Louis was approached in a local charity shop. Louis made himself available for the interview immediately. I met Samira at Place de la République. The (male) *sans-papiers* who had gathered there pointed me to Samira, mostly because she was a female, and because, on other occasions, she had acted as the spokesperson of the Coordination Sans Papiers 75. The official coordinator provided me with photos of the public events that had been organised during the pandemic, but he delegated Samira to speak to me.

^7^ The second round of elections was held on 28 June 2020.

^8^ The translation from French to English is mine.

^9^ The concept of ‘the uncounted’ ( Rancière, 1999: 38) is perhaps the most fitting for this context. The ‘uncounted’ are, for Rancière, the invisible, the marginalised, those not counted as part of the community.

^10^ Samira is a fictional name, as are all the other names used in this article. However, in the process of “renaming” the person, I have taken into consideration their gender and nationality. Samira is a Tunisian, who consented to talk to me, on 16 December 2022, at Place de la République, during the Friday weekly gathering. The interview was conducted in French but, while listening to her, I formulated some extra questions in English rather than in French, as they came to me spontaneously, and I was aware that Samira could understand English.

^11^ It goes without saying that the politics of control and surveillance against *sans-papiers*, as well as the criminalisation of any act of solidarity toward them, pre-existed COVID-19 (see Dadusc & Mudu, 2022; López-Sala & Barbero, 2019).

^12^ As of 2022, after six long years in France, her legal status has been regularised.

^13^ Lejla is a volunteer who has been working with Care4Calais since The Jungle was demolished and many unaccompanied minors came to Paris in 2016. I approached her during my fieldwork in Paris through a network of scholars who have been engaged, academically and voluntarily, with *sans-papiers* and asylum-seekers. She is from Sarajevo and became a refugee when she was 14. Our conversation took place on 13 December 2022. It was primarily in English, with only a few words in French.

^14^ Unless otherwise stated, the contents of this section come from my long conversation with Lejla.

^15^ She noted that unaccompanied minors tend to look older than they are. In fact, she looked 40 when she left Sarajevo, when she was only 14.

^16^ DEMIE stands for *dispositif d’evaluation des mineurs isolés étrangers* (assessment system for unaccompanied foreign minors). Since July 2022, France Terre d’Asile has been in charge of the age assessment process.

^17^ Human Rights Watch has been especially critical of Red Cross activities, and the title of its report is telling: Like a Lottery ( 2018).

^18^ La Goutte d’Or is a multi-ethnic and multi-linguistic neighbourhood located in the 18th arrondissement. Traditionally, it has been a working-class area, and since the post-WWII period, one with a high concentration of residents from Maghreb and Sub-Sahara. This area is especially known for Saint-Bernard de la Chapelle Church, which became a sanctuary for some 300 *sans-papiers* in June 1996. Next to the church, the organisation Salle Saint-Bruno is active in the neighbourhood, and particularly sensitive to issues of equality, rights and refugee inclusion. On 15 December 2022, while carrying out my fieldwork in Paris, I attended the meeting organised in light of the forthcoming public event to be held on Sunday, 18 December, on the occasion of International Migrants Day, under the slogan ‘ *Solidarité, liberté, égalité, papiers*’.

^19^ The conversation with Louis took place on 15 December 2022, mostly in English. I approached Louis, a few days before, at a local thrift store, in Rue Léon, where he was doing voluntary work. He agreed to share his experience.

^20^ The Brigades de Solidarité Populaire was still active at the time of my fieldwork, in La Goutte d’Or, mostly through a local library, small shops selling used items and arranging food distribution drives.

^21^ ‘A political action within a political conscience. It is legitimate.’ I left the French here as to highlight that those words were expressed in French and not in English.

^22^ The issue of whether to conform to rules or choose to assist people in need—despite the legal restrictions—did not only emerge during the COVID-19 pandemic. Similar cases that come to mind are the many ways in which “border people” and NGOs help and assist (unauthorised) migrants despite the many restrictions and penalties introduced by governments ( Puggioni, 2024). However, their actions—in the name of solidarity, humanity and a sense of duty—incorporate a key political component that the mobility practices considered here did not. However, their response-ability is also the result of an impossible (ethical) choice.
