# Supplementary material for: COVID-19 and (ir)responsible (im)mobility: Reading counter-practices through Levinas and Derrida
Source: Open Res Eur. 2024 Oct 7;4:2. Originally published 2024 Jan 3. [Version 2] doi: 10.12688/openreseurope.16686.2 (PMC11487237; doi:10.12688/openreseurope.16686.2)
Supplement: Peer review history

# Reviewer report: Reviewer response for version 2

**DOI:** 10.21956/openreseurope.20076.r44905

**Published:** 2025-11-04

**Authors:** Gaja Maestri

**Affiliations:** 1 Aston University, Birmingham, England, UK

I'm satisfied with the revisions.

Is the case presented with sufficient detail to be useful for teaching or other practitioners?

Partly

Is the work clearly and accurately presented and does it cite the current literature?

Partly

If applicable, is the statistical analysis and its interpretation appropriate?

Not applicable

Are all the source data underlying the results available to ensure full reproducibility?

Yes

Are the conclusions drawn adequately supported by the results?

Yes

Is the background of the case’s history and progression described in sufficient detail?

Yes

Reviewer Expertise:

Social movements and collective action; migrant solidarity

I confirm that I have read this submission and believe that I have an appropriate level of expertise to confirm that it is of an acceptable scientific standard.

# Reviewer report: Reviewer response for version 2

**DOI:** 10.21956/openreseurope.20076.r44904

**Published:** 2024-10-24

**Authors:** Carina van de Wetering, Thomas de Jong

**Affiliations:** 1 Institute of Political Science, Leiden University, Leiden, The Netherlands; 2 Netherlands Public Prosecution Service, The Hague, The Netherlands

We would like to thank the author for addressing our concerns. The co-reviewer and I just have a few additional points left on the basis of the revisions:  

 -We would like to recommend a change in wording in the following passage to stay in keeping with the rest: "It is not about following orders, norms, and dominant practices but about breaking away from them" (p. 6). However, it is not about breaking away from dominant practices for Derrida but about reinventing them; it is not a fight against hegemony which this sentence could imply.

 -The article mentions in the conclusion that the respondents did not use the concept of solidarity. In fact, one of the respondents named Louis did refer to solidarity as an important aspect for his engagement (p. 11).

 -The article mentions civic responsibility, charity and solidarity nearing the conclusion (p. 11), but it is not clear what the difference between civic responsibility and solidarity is. Moreover, the article refers to collective and individual responsibility at the start (p. 3), but does not come back to these concepts. This would need some finetuning.

Is the case presented with sufficient detail to be useful for teaching or other practitioners?

Partly

Is the work clearly and accurately presented and does it cite the current literature?

Partly

If applicable, is the statistical analysis and its interpretation appropriate?

Not applicable

Are all the source data underlying the results available to ensure full reproducibility?

Yes

Are the conclusions drawn adequately supported by the results?

Partly

Is the background of the case’s history and progression described in sufficient detail?

Yes

Reviewer Expertise:

Van de Wetering: International Relations, US foreign policy, critical security studies, and discourse theory. De Jong: legal philosophy, ethics, existentialism, and criminal law.

We confirm that we have read this submission and believe that we have an appropriate level of expertise to confirm that it is of an acceptable scientific standard.

# Reviewer report: Reviewer response for version 2

**DOI:** 10.21956/openreseurope.20076.r44906

**Published:** 2024-10-17

**Authors:** Arleen Ionescu

**Affiliations:** 1 Universitatea de Vest din Timisoara, Timișoara, Timiș County, Romania

The author has made significant changes which makes her work more coherent, stylish and solid.

Is the case presented with sufficient detail to be useful for teaching or other practitioners?

Yes

Is the work clearly and accurately presented and does it cite the current literature?

Yes

If applicable, is the statistical analysis and its interpretation appropriate?

I cannot comment. A qualified statistician is required.

Are all the source data underlying the results available to ensure full reproducibility?

Yes

Are the conclusions drawn adequately supported by the results?

Partly

Is the background of the case’s history and progression described in sufficient detail?

Yes

Reviewer Expertise:

critical theory, deconstruction, trauma, memory studies, refugees, ethics, etc.

I confirm that I have read this submission and believe that I have an appropriate level of expertise to confirm that it is of an acceptable scientific standard.

# Reviewer report: Reviewer response for version 1

**DOI:** 10.21956/openreseurope.18019.r40688

**Published:** 2024-06-05

**Authors:** Carina van de Wetering, Thomas de Jong

**Affiliations:** 1 Institute of Political Science, Leiden University, Leiden, The Netherlands; 2 Netherlands Public Prosecution Service, The Hague, The Netherlands

Although the COVID-19 pandemic is often considered as a crisis confined to the past, the ethical questions that emerged are still profound and stimulating enough to give us a pause. Puggioni (2024) discusses one of the major ethical concerns through the overarching aim of the (ir)responsibility to assist and make visible the needs of very marginalized people, in particular asylum-seekers, refugees and *sans-papiers*, in a state of mobility restrictions. In order to demonstrate this, she makes use of three rich case studies that are very instructive for her study. However, as we will pinpoint here below, this could have been embedded more within the current literature and her own discussion of Jacques Derrida.

 The author argues how the literature on civic attitudes under COVID-19 restrictions has dealt with political activism and the democratic state, but rather not with the issue of what counts as responsible acts (Puggioni, 2024, p. 3). The article mentions that the research findings hopefully “encourage a (theoretical) debate on questions of *ethical *and *civic *responsibility during a life-threatening emergency” (Puggioni, 2024, p. 4). In that light, we would like to refer to a special issue, entitled: “Solidarity and Covid-19” which sparked this international debate by reflecting on the implications of such an emergency for our solidarity stance (Van den Berge, Duff and Veraart, 2021). As one of the contributors to this special issue, we asked specifically what the meaning and scope of solidarity is under pandemic conditions, by drawing on insights of Levinas and Derrida, similar to the current author. We characterized solidarity as “the primary responsibility we bear for the other, to which the other as ‘wholly other’ invites me” (De Jong and Van de Wetering, 2021, p. 152). This implies going through an ordeal of undecidability, that is demonstrated in a few examples of caregivers.

 However, a strength of Puggioni’s article is that it brings to attention harrowing cases of marginalized and often disregarded groups, to illustrate the counter-practices of volunteers who were trying to help by engaging in solidarity actions. These cases are a striking testament to the positive response that is given to the appeal of the face of the other, which is in keeping with the theoretical elaboration. For example, one interviewee notes how she was compelled to act by seeing the injustice and making a political statement to “correct it”, while the other saw it as responsible citizenship (Puggioni, 2024, pp. 7-8). In discussing these, the author highlights here the “ability to react” because of a “sense of responsibility” (Puggioni, 2024, p. 7). This seems to come back to the ethical and civic responsibility, although the author makes that point not explicitly.

 Furthermore, the Levinasian conception of responsibility is somewhat underdeveloped. The author states that responsibility emerges, because the self feels obliged to respond, thereby suggesting that responsibility stems from or is guided by a feeling of obligation (Puggioni, 2024, p. 9). This is not in accord with Levinas, who emphatically insists that I am responsible for the other “despite myself” ( *malgré moi*). It is a responsibility that can in no way be reduced to feelings of obligation or sympathy – i.e. the self - but comes from the other and breaks through the totality of the self, before one can feel, want or think. So, it is not as much the self that “creates an ethical relationship” but the other (Puggioni, 2024, p. 9).

 The article does provide an elucidating discussion of the ethical implications of responsibility, through Derrida’s notion of undecidability. However, the exact link between the case and theory remains somewhat unclear as the theoretical discussion comes to an abrupt stop. It would help to better tie together the theory and the cases. Moreover, the article addresses several aims at the start about what it means to act in a irresponsible way during a life-threatening emergency, whether it is irresponsible in regard to mobility restrictions  and how it can be judged, but it does not satisfactorily wrap up all these questions because of the open-ended conclusion. More importantly, for Levinas and Derrida, our responsibility toward the other is about a unique responsibility in a singular situation, as also reflected in the ordeal of undecidability. Even though there is an obligation to respond to the need of the other, you are free to assume what to do. In every situation one has to reinvent for oneself what responsibility demands. Thus, it then becomes unclear how we can come up with particular criteria to evaluate that. This should be reconsidered by the author.

 All in all, we the think the article should be approved of, but with reservations.

Is the case presented with sufficient detail to be useful for teaching or other practitioners?

Partly

Is the work clearly and accurately presented and does it cite the current literature?

Partly

If applicable, is the statistical analysis and its interpretation appropriate?

Not applicable

Are all the source data underlying the results available to ensure full reproducibility?

Yes

Are the conclusions drawn adequately supported by the results?

Partly

Is the background of the case’s history and progression described in sufficient detail?

Yes

Reviewer Expertise:

Van de Wetering: International Relations, US foreign policy, critical security studies, and discourse theory. De Jong: legal philosophy, ethics, existentialism, and criminal law.

We confirm that we have read this submission and believe that we have an appropriate level of expertise to confirm that it is of an acceptable scientific standard, however we have significant reservations, as outlined above.

## References

[1] : Welcoming the Other in a Pandemic Society. *Netherlands Journal of Legal Philosophy* .2021;50(2) : 10.5553/NJLP/221307132021050002005 151-167 10.5553/NJLP/221307132021050002005

[2] : Solidarity and COVID-19: An Introduction. *Netherlands Journal of Legal Philosophy* .2021;50(2) : 10.5553/NJLP/221307132021050002001 109-119 10.5553/NJLP/221307132021050002001

# Response

**Published:** 2024-09-14

**Authors:** Raffaela Puggioni

**Affiliations:** School of International Affairs, OP Jindal Global University, Sonipat, Haryana, India

**Carina van de Wetering, Institute of Political Science, Leiden University, Leiden, The Netherlands** Thank you for accepting to make comments on the manuscript. I have divided your comments into three (thematic) queries, as specified below:

1. **Theoretical part: ethics in Levinas and Derrida**

The literature on ethics has been expanded, and more attention given to Levinas on the meaning of ethical relations. I have strengthened the part on ethical relations in order to better highlight the questions that ethics raises and how they are closely connected with the debate during the pandemic. I have incorporated great part of the articles included in the special issue suggested (Van den Berge, Duff and Veraart, 2021), and I very much appreciated the suggestion. I have not incorporated the part on solidarity as this concept was not really used by my respondents. But I have extensively drawn from that special issue in reference to the general debate on ethics and conformity to rules. I have reformulated a few sentences and removed the concept of ‘feeling’ and highlighted more the concept of ‘ability-to-respond’. Given the greater emphasis on Levinas, I have reformulated the title of the article and added ‘Levinas’.

1. **Connection between theory and practice**

This has been done in two steps: 1) adding a section on ethics and ethical dilemma and 2) re-writing the conclusion in which the two are better connected.

1. **Conclusion**

The concluding section of the article has been re-written. I have better connected the theoretical part with the personal narratives. Given the inclusion of the work of Derrida, I have not really judged counter-practices as responsible or irresponsible, but highlighted that a clear line is impossible, at least from an ethical perspective.

# Reviewer report: Reviewer response for version 1

**DOI:** 10.21956/openreseurope.18019.r38644

**Published:** 2024-05-25

**Authors:** Arleen Ionescu

**Affiliations:** 1 Universitatea de Vest din Timisoara, Timișoara, Timiș County, Romania

The article is generally well structured and well written. It has several innovative ideas and engages with very interesting materials. I think what it lacks in its current state is more engagement with the theoretical background of ethics, in particular Derrida's notion of ethics (which otherwise is introduced very late in the article- a suggestion is to introduce the connection in the paragraph where she lists her research questions because the title does mention a "reading" through Derrida). Also, Derrida's name should appear in the keywords. The author explains well that Derrida's understanding of ethics is related to Levinas's ethics and uses both primary sources (Derrida's *Force of Law,* *The Gift of Dearth*, *On the Name*) and several secondary sources not necessarily the most relevant, I would say, but this is of course the author's choice. Yet, the author ignores important texts by Derrida such as "Hos(ti)pitality" or *On Hospitality* where he engaged with how the Other is welcomed and the notion of "sans-papiers". Also, Derrida's "Paper or Myself, You Know..." might offer interesting insights into the notion of "sans-papiers" (see https://www.jstor.org/stable/i40128694). Derek Attridge's *Reading and Responsibility* (Edinburgh UP, 2010) might also provide the author with some other thoughts on responsibility that can be useful as a theoretical background for her findings.

 I am not very sure that asserting "This was done using both Google Scholar and Google search." shows that the author used proper research methods. Anybody can do documentation on Google search, but don't researchers use proper databases where they can find proper academic articles? 

 The author also mentions: "In terms of the research framework, I adopted a grounded theory approach, even if only three interviews have been carried out." Shouldn't we be told why she decided to do only three interviews and whether they provided her with enough material to draw her conclusions? 

 Another thing which is quite striking is that the author cites extremely many articles from her own work. I do understand that in order to avoid self-plagiarism when we engage with our own (previous) work we need to use quotation marks and proper referencing, yet it seems to me that the author overdoes it a bit, since there are 9 titles in the Reference List from her own work and I am not 100% sure that all of them are necessary or relevant to this theme. From all works in the Biblio, her own titles are the most numerous. It looks too much as if the author aims to score as many points as possible from self-citation, which is not ethical.

 I think the concluding remarks should not end on another series of questions. We should be provided with answers rather than questions at the end of an article, which shows that the author achieved her tasks (these were formulated as a series of questions at the beginning of the article where she announced us what she will do).

Is the case presented with sufficient detail to be useful for teaching or other practitioners?

Yes

Is the work clearly and accurately presented and does it cite the current literature?

Yes

If applicable, is the statistical analysis and its interpretation appropriate?

I cannot comment. A qualified statistician is required.

Are all the source data underlying the results available to ensure full reproducibility?

Yes

Are the conclusions drawn adequately supported by the results?

Partly

Is the background of the case’s history and progression described in sufficient detail?

Yes

Reviewer Expertise:

critical theory, deconstruction, trauma, memory studies, refugees, ethics, etc.

I confirm that I have read this submission and believe that I have an appropriate level of expertise to confirm that it is of an acceptable scientific standard, however I have significant reservations, as outlined above.

# Response

**Published:** 2024-09-14

**Authors:** Raffaela Puggioni

**Affiliations:** School of International Affairs, OP Jindal Global University, Sonipat, Haryana, India

**Response to Dr** **Arleen Ionescu, Shanghai Jiao Tong University, Shanghai, China** Thank you for accepting to make comments on the manuscript. I have divided your comments into four (thematic) queries, as specified below:

1. **Methodology**

The issue of methodology was also raised by the first reviewer. I have added a few extra information of my respondents. This has been incorporated in the endnote no. 5, in which I clarified how I came across to them. Endnote no. 3 clarifies the questions that have been raised. It should be noted that I have not introduced any changes in this note. Regarding the number of interviews, I have recognised the limited impact, but the key aim of my research was not to collect data in order to quantify the widespread of these (mobility) practices, which mostly go unnoticed. I initially submitted the article as a theoretical article and not as a case study (which was a suggestion of the editorial team), as I personally believe that three interview do not make a case study, but should be seen more as three (significant) examples on which to reflect. Regarding the use of Google Scholar (for academic works) and Google search (for newspaper articles), I do not really believe that it should be problematic *per se*. It would be a problem if the academic references used are irrelevant, inadequate or non-academic, which I do not believe is the case. If I can be honest, I used Google Scholar as, in comparison to the LUISS’s library search, it was more helpful when searching through key words.

1. **Derrida’s work**

I have expanded the ethical discussion by incorporating an extra section. The aim was to better connect the debate on responsibility with ethics and the personal narratives used. This was a major shortcoming that all the reviewers pointed out. Regarding your suggestions on Derrida, I greatly appreciated your suggestion on adding Derek Attridge's *Reading and Responsibility* (Edinburgh UP, 2010). I did not come across to his work before. His work highlighted a few points that I overlooked, which I have incorporated. I have not directly engaged, in this article, with hospitality and sans-papiers as I was more interested in expanding on ethics and responsibility. However, rather than expanding on Derrida’s ethics, I have expanded on Levinas’ work, which in the original version was very limited. I have not included Derrida among the key words, as his name is already used in the title. 

1. **References to my work**

I personally do not think that to include one’s work in the reference is ‘unethical’, if what is included well connects with the manuscript’s overall theme. In light of the comments, I have removed all the reference on my previous works on asylum-seekers/undocumented. I do believe that the remaining references should be kept, for the following reasons: **1)** one article deal with COVID-19 counter-practice in Italy, part of the very same Horizon 2020 project; **2)** one article is on ethics, solidarity and protests in the Roya Valley (despite restrictions), which the first reviewer for instance brought in. Keeping this reference is a way of recognising that I have already considered some commonalities; **3)** two references are not strictly speaking references. They refer to the link to the data set. For some reasons, the editorial team made me to include them twice: in the data availability section and again in the reference list. So, 4 references in total and two data set under my name.

1. **Concluding remarks**

The concluding section of the article has been re-written. I have in particular connected the theoretical with the examples introduced and highlighted why it is important to add an ethical perspective to the general debate on (ir)responsibility during COVID-19.

# Reviewer report: Reviewer response for version 1

**DOI:** 10.21956/openreseurope.18019.r38647

**Published:** 2024-04-22

**Authors:** Gaja Maestri

**Affiliations:** 1 Aston University, Birmingham, England, UK

Thank you for inviting me to review this article. which explores practices of mobility during the Covid19 pandemic in the city of Paris. Based on the analysis of three interviews, it highlights the ambiguity of what constitute (ir)responsible citizenship in times of emergency. The article is presented clearly and offers some interesting empirical evidence, applying Derrida's concept of responsibility to the acts of mobility of one undocumented migrant and two volunteers who supported vulnerable migrants during the pandemic.

 Whilst I think that the paper contributes to debates around mobility and civic responsibility during the pandemic through three empirical examples, I believe that it also presents some weaknesses.

 First, the methodological approach of the research is not entirely clear. Whilst the author frankly discusses the unpredictability of the research process and explains the changes to the initial research design, it is not clear who participants to the research are and what the interviews focused on. While reading the article, it appears that the 3 participants are one undocumented migrant, one (expat?) volunteer, and one French citizen, but it would be important to understand why the researcher chose these three profiles exactly. Moreover, basing an article on only 3 interviews is clearly limited. Nevertheless, since there are no claims to generalisation and representativeness, and the research aim is to take a deep look into the individual narratives, this number could still be acceptable. However, very little details are given of the participants, especially of the first (Samira). I believe that to strengthen a very small-n qualitative fieldwork, more details would be needed.

 Secondly, whilst the article offers an extremely detailed section on the measures adopted by the French government during the Covid-19 pandemic, it does not clearly situates its contribution within academic literature. The concept of responsibility is brought in at the end, without really clarifying why. I understand that the main question of the article is what constitute a responsible/civic act during the pandemic, but the question is not strongly justified apart from saying that responsibility has attracted little academic attention. I would find it more convincing if the author could first review the literature on mobility during the pandemic, and then show that this literature has overlooked the question of how certain types of mobility were justified in a moment of mobility restriction.

 Following on from my previous point, the author also mentions the literature on protests and political activism (in the Introduction) but does not really engage with it. On the contrary, I think this would have been particularly fruitful for the research. Similar to those who decided to move during the pandemic, even though that was deemed illegal and irresponsible, scholarly works on social movements have focused on activism and civil disobedience or non-violent direct action and how these are justified as responsible even though they are legally contentious. To me, what the author is considering is in fact a case of people who decide to break the law to answer a greater cause -- i.e., helping the most vulnerable. How is this different from the act of volunteers and activists who support undocumented migrants in crossing borders? I'm thinking, among others, about the case of Cédric Herrou who was initially sued for breaking the law (and was later found not guilty) but for also doing something that many support, i.e. defending and helping migrants. I think that a wider discussion on the tension between legality and responsibility would have offered a stronger theoretical approach and would have helped justify more clearly the research focus on (ir)responsibility.

 On a very minor note, a p.3 and p.4 "the followings" should be "the following".

Is the case presented with sufficient detail to be useful for teaching or other practitioners?

Partly

Is the work clearly and accurately presented and does it cite the current literature?

Partly

If applicable, is the statistical analysis and its interpretation appropriate?

Not applicable

Are all the source data underlying the results available to ensure full reproducibility?

Yes

Are the conclusions drawn adequately supported by the results?

Yes

Is the background of the case’s history and progression described in sufficient detail?

Yes

Reviewer Expertise:

Social movements and collective action; migrant solidarity

I confirm that I have read this submission and believe that I have an appropriate level of expertise to confirm that it is of an acceptable scientific standard, however I have significant reservations, as outlined above.

# Response

**Published:** 2024-09-14

**Authors:** Raffaela Puggioni

**Affiliations:** School of International Affairs, OP Jindal Global University, Sonipat, Haryana, India

**Response to Dr. Gaja Maestri, Aston University, Birmingham, England, UK** Thank you for accepting to make comments on the manuscript. I have divided your comments into four (thematic) queries, as specified below:

1. **Methodology**

I have added a few extra information of my respondents. This has been incorporated in the endnote no. 5, in which I clarified how I came across to them. Endnote no. 3 clarifies the questions that have been raised. It should be noted that I have not introduced any changes in this note.  

**2. Wider debate on (im)mobility, (ir)responsibility and ethics**          I have re-written the introduction, added a new section on ‘Emergency and the duty to protect’, and expanded on the concept of ethics, and ethical subject. The section on ‘emergency and duty to protect’ offers a general overview of the debate on the ‘duty to protect’ oneself and others by respecting COVID-19 exceptional norms. I have especially highlighted the debate that emerged at that time: on the one hand, the ‘duty’ and legal obligations to respect rules and, on the other, its opposite perspective, i.e. the restrictions imposed were not only illiberal but they also jeopardised individual freedom. This extra section not only helps connect my article with the wider debate on COVID-19 and (ir)responsibility, but it also connects with the ethical responses that emerged in the interviews that I carried out.

**3. Literature on protests and political activism**          Although I included some mentioned protests and political activism, and included some references, I decided not to engage on them for two reasons. Firstly, the key aim of the article is on ethics, and ethical perspective, which is distinctively personal. Secondly, I wanted to keep separate the ethical from the political, which political activism involves.

**4. Commonalities with volunteers and activists supporting undocumented migrants. **          There are certainly some similarities, and I have added an endnote on this, no. 21. I did not specifically made any comparison, for the following reasons: 1) the case of Roya Valley was articulated upon the concept of ‘solidarity’, a concept that did not emerge in the interviews carried out; 2) the limited number of interviews carried out does not (properly) allow for any comparison; 3) the political component—i.e. make Italian and French government to revisit the ‘criminalisation of solidarity’—is missing in this article.
